# Supplementary material for: Comprehensive Cancer-Predisposition Gene Testing in an Adult Multiple Primary Tumor Series Shows a Broad Range of Deleterious Variants and Atypical Tumor Phenotypes
Source: Am J Hum Genet. 2018 Jun 14;103(1):3–18. doi: 10.1016/j.ajhg.2018.04.013 (PMC6037202; doi:10.1016/j.ajhg.2018.04.013)
Supplement: Document S1. Figures S1 and S2 and Tables S2 and S7 [file mmc1.pdf]

The American Journal of Human Genetics, Volume *103*

Supplemental Data

Comprehensive Cancer-Predisposition Gene Testing in an Adult Multiple Primary Tumor Series Shows a Broad Range of Deleterious Variants and Atypical Tumor Phenotypes

James Whitworth, Philip S. Smith, Jose-Ezequiel Martin, Hannah West, Andrea Luchetti, Faye Rodger, Graeme Clark, Keren Carss, Jonathan Stephens, Kathleen Stirrups, Chris Penkett, Rutendo Mapeta, Sofie Ashford, Karyn Megy, Hassan Shakeel, Munaza Ahmed, Julian Adlard, Julian Barwell, Carole Brewer, Ruth T. Casey, Ruth Armstrong, Trevor Cole, Dafydd Gareth Evans, Florentia Fostira, Lynn Greenhalgh, Helen Hanson, Alex Henderson, Jonathan Hoffman, Louise Izatt, Ajith Kumar, Ava Kwong, Fiona Lalloo, Kai Ren Ong, Joan Paterson, Soo-Mi Park, Rakefet Chen-Shtoyerman, Claire Searle, Lucy Side, Anne-Bine Skytte, Katie Snape, Emma R. Woodward, NIHR BioResource Rare Diseases Consortium, Marc D. Tischkowitz, and Eamonn R. Maher

Supplemental Data

Supplemental Figures

Figure S1 - SNV/indel workflow

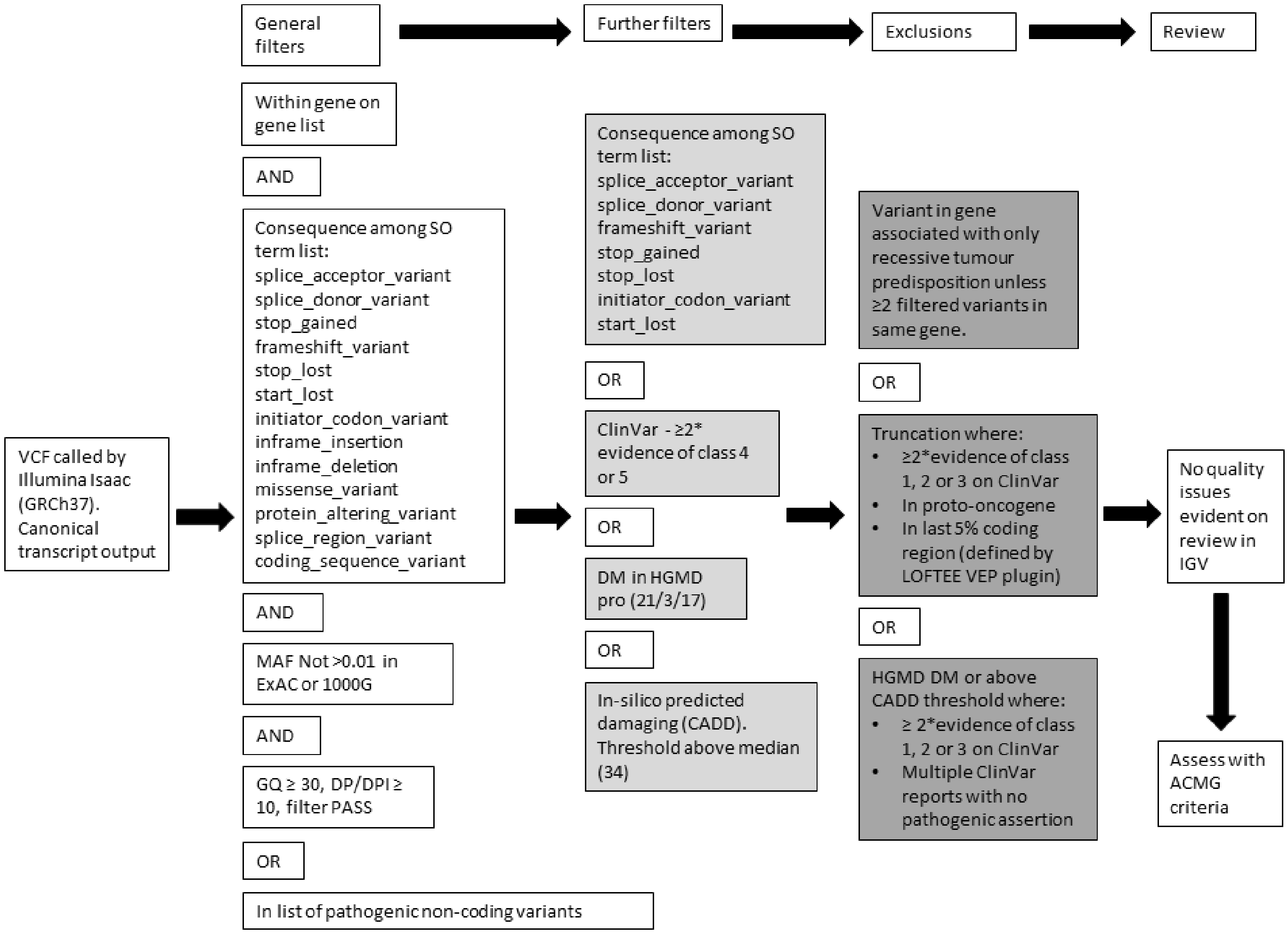

Figure S2 - Structural variant workflow

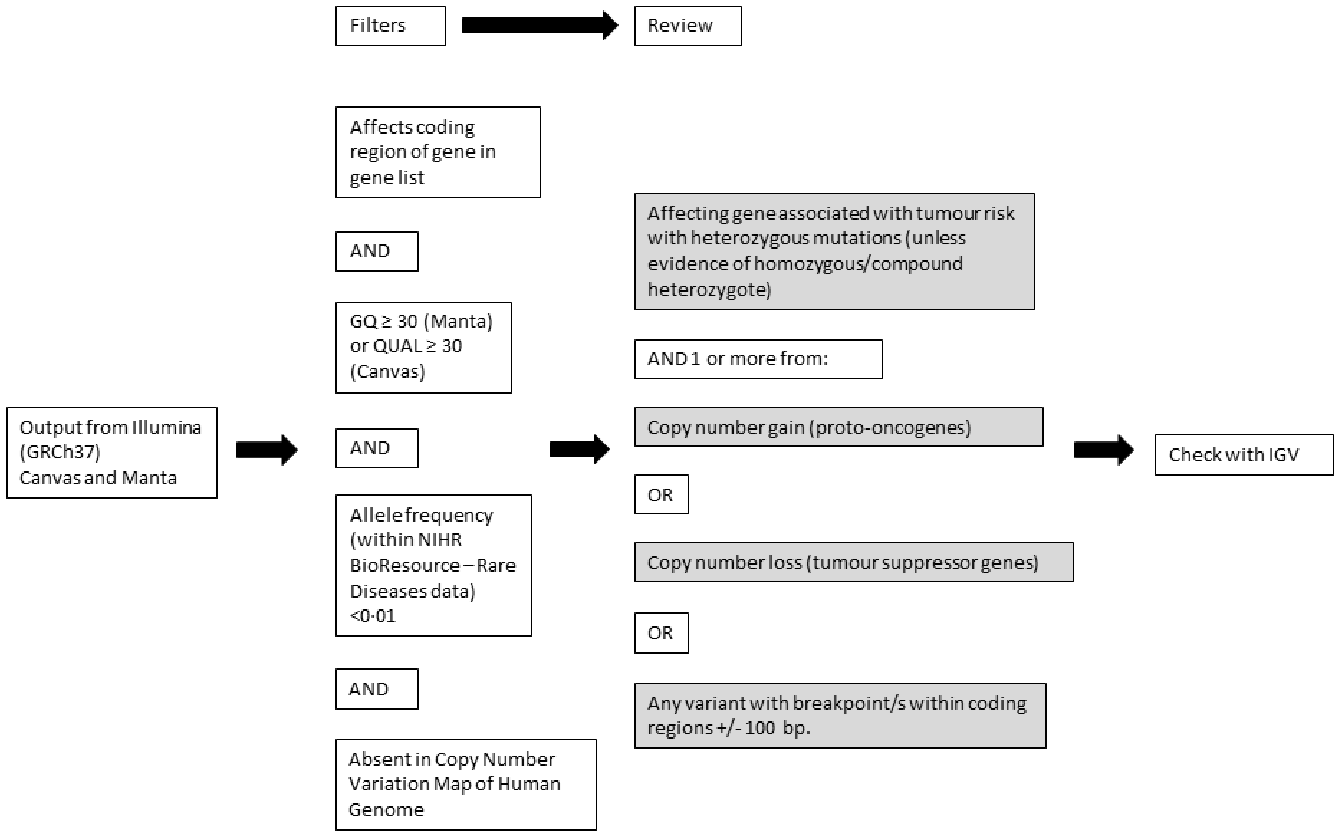

Supplemental tables

Table S2 – Genes sequenced by Illumina TruSight Cancer panel on list of 83 analyzed genes

AIP	*DICER1*	*MLH1*	*RUNX1*
ALK	*EGFR*	*MSH2*	*SDHAF2*
APC	*EPCAM*	*MSH6*	*SDHB*
ATM	*ERCC2*	*MUTYH*	*SDHC*
BAP1	*ERCC3*	*NF1*	*SDHD*
BMPR1A	*ERCC4*	*NF2*	*SMAD4*
BRCA1	*ERCC5*	*PALB2*	*SMARCB1*
BRCA2	*EXT1*	*PHOX2B*	*STK11*
BRIP1	*EXT2*	*PMS2*	*SUFU*
CDC73	*FH*	*PRKAR1A*	*TMEM127*
CDH1	*FLCN*	*PTCH1*	*TP53*
CDK4	*GATA2*	*PTEN*	*TSC1*
CDKN2A	*HNF1A*	*RAD51C*	*TSC2*
CEBPA	*KIT*	*RAD51D*	*VHL*
CHEK2	*MAX*	*RB1*	*WT1*
CYLD	*MEN1*	*RET*	*XPA*
DDB2	*MET*	*RHBDF2*	*XPC*

Table S7 – Comparison of tumor combinations in series vs East Anglia Registry 2009-2014.

Cancer A	Cancer B	Data source	Combination count	% total	Combination type makes up ≥1% total in MPT and EA	Difference in proportion of total MPT vs EA (%)	χ^2 p-value (MPT vs EA)
Breast	Colorectal	MPT	29	5.5	YES	2.7	0.02145
Breast	Ovary	MPT	23	4.4	YES	2.5	0.01786
Breast	Endometrium	MPT	20	3.8	YES	0.1	0.8852
Breast	NMSC	MPT	19	3.6	YES	-10.2	<0.00001
Breast	Thyroid	MPT	19	3.6	NO	3.2	0.00007
Breast	Hem Lymphoid	MPT	18	3.4	YES (note different classification MPT vs EA)	2	0.02594
Endometrium	Ovary	MPT	17	3.3	YES	1.6	0.1073
Breast	Melanoma	MPT	14	2.7	YES	0.6	0.5397
Breast	CNS Meningioma	MPT	7	1.3	NO (Classified as "Brain" in EA)	0.9	0.06944
Breast	Kidney	MPT	6	1.1	NO	0.9	0.04323
Breast	Lung	MPT	6	1.1	NO	0	0.252
Melanoma	Thyroid	MPT	6	1.1	NO	0.7	0.1204
Breast	NMSC	EA	79	13.8	NO	N/A	N/A
Melanoma	NMSC	EA	64	11.1	NO	N/A	N/A
Hematological	NMSC	EA	29	5.1	NO	N/A	N/A
NMSC	Prostate	EA	26	4.5	NO	N/A	N/A
Breast	Uterus	EA	21	3.7	NO	N/A	N/A
Breast	Melanoma	EA	19	3.3	NO	N/A	N/A
Breast	Ovary	EA	11	1.9	NO	N/A	N/A
Prostate	Renal tract	EA	10	1.7	NO	N/A	N/A
Ovary	Uterus	EA	10	1.7	NO	N/A	N/A
NMSC	Thyroid	EA	9	1.6	NO	N/A	N/A
Breast	Hematological	EA	8	1.4	NO	N/A	N/A
Breast	Colorectal	EA	16	2.8	NO	N/A	N/A
Colorectal	NMSC	EA	14	2.4	NO	N/A	N/A
Breast	Cervix	EA	7	1.2	NO	N/A	N/A
Hematological	Prostate	EA	6	1.0	NO	N/A	N/A
NMSC	Ovary	EA	6	1.0	NO	N/A	N/A
NMSC	Uterus	EA	6	1.0	NO	N/A	N/A

Only tumors occurring before age 60 included for consistency with registry data as obtained.

MPT – Multiple primary tumor series (data analyzed in this study). EA – East Anglia Registry Data.

CNS – Central nervous system, Hem – Hematological, NMSC - Non-melanoma skin cancer (includes basal cell carcinoma and squamous cell carcinoma).

Supplemental References

1. Rahman, N. (2014). Realizing the promise of cancer predisposition genes. Nature 505, 302–308.

2. Finn, R.D., Coggill, P., Eberhardt, R.Y., Eddy, S.R., Mistry, J., Mitchell, A.L., Potter, S.C., Punta, M., Qureshi, M., Sangrador-Vegas, A., et al. (2015). The Pfam protein families database: towards a more sustainable future. Nucleic Acids Res. 44, D279–D285.

3. James Kent, W., Sugnet, C.W., Furey, T.S., Roskin, K.M., Pringle, T.H., Zahler, A.M., and Haussler, D. (2002). The human genome browser at UCSC. Genome Res. 12, 996–1006.
