# Supplementary material for: Comprehensive Cancer-Predisposition Gene Testing in an Adult Multiple Primary Tumor Series Shows a Broad Range of Deleterious Variants and Atypical Tumor Phenotypes
Source: Am J Hum Genet. 2018 Jun 14;103(1):3–18. doi: 10.1016/j.ajhg.2018.04.013 (PMC6037202; doi:10.1016/j.ajhg.2018.04.013)
Supplement: Document S2. Article plus Supplemental Data [file mmc7.pdf]

Comprehensive Cancer-Predisposition Gene Testing in an Adult Multiple Primary Tumor Series Shows a Broad Range of Deleterious Variants and Atypical Tumor Phenotypes

James Whitworth,[1] Philip S. Smith,[1] Jose-Ezequiel Martin,[1] Hannah West,[1] Andrea Luchetti,[1] Faye Rodger,[1] Graeme Clark,[1] Keren Carss,[2,3] Jonathan Stephens,[2,3] Kathleen Stirrups,[2,3] Chris Penkett,[2,3] Rutendo Mapeta,[2,3] Sofie Ashford,[2] Karyn Megy,[2,3] Hassan Shakeel,[1] Munaza Ahmed,[4,5] Julian Adlard,[6] Julian Barwell,[7] Carole Brewer,[8] Ruth T. Casey,[1] Ruth Armstrong,[1,2,9] Trevor Cole,[10] Dafydd Gareth Evans,[11] Florentia Fostira,[12] Lynn Greenhalgh,[13] Helen Hanson,[14] Alex Henderson,[15] Jonathan Hoffman,[10] Louise Izatt,[16] Ajith Kumar,[5] Ava Kwong,[17,18,19] Fiona Lalloo,[11] Kai Ren Ong,[10] Joan Paterson,[9] Soo-Mi Park,[1,2,9] Rakefet Chen-Shtoyerman,[20,21] Claire Searle,[22] Lucy Side,[4,5] Anne-Bine Skytte,[23] Katie Snape,[14] Emma R. Woodward,[11] NIHR BioResource Rare Diseases Consortium,[2] Marc D. Tischkowitz,[1,2] and Eamonn R. Maher[1,2,*]

Multiple primary tumors (MPTs) affect a substantial proportion of cancer survivors and can result from various causes, including inherited predisposition. Currently, germline genetic testing of MPT-affected individuals for variants in cancer-predisposition genes (CPGs) is mostly targeted by tumor type. We ascertained pre-assessed MPT individuals (with at least two primary tumors by age 60 years or at least three by 70 years) from genetics centers and performed whole-genome sequencing (WGS) on 460 individuals from 440 families. Despite previous negative genetic assessment and molecular investigations, pathogenic variants in moderate- and high-risk CPGs were detected in 67/440 (15.2%) probands. WGS detected variants that would not be (or were not) detected by targeted resequencing strategies, including low-frequency structural variants (6/440 [1.4%] probands). In most individuals with a germline variant assessed as pathogenic or likely pathogenic (P/LP), at least one of their tumor types was characteristic of variants in the relevant CPG. However, in 29 probands (42.2% of those with a P/LP variant), the tumor phenotype appeared discordant. The frequency of individuals with truncating or splice-site CPG variants and at least one discordant tumor type was significantly higher than in a control population ($\chi^2 = 43.642$; $p \leq 0.0001$). 2/67 (3%) probands with P/LP variants had evidence of multiple inherited neoplasia allele syndrome (MINAS) with deleterious variants in two CPGs. Together with variant detection rates from a previous series of similarly ascertained MPT-affected individuals, the present results suggest that first-line comprehensive CPG analysis in an MPT cohort referred to clinical genetics services would detect a deleterious variant in about a third of individuals.

Introduction

Inherited cancer-predisposition syndromes account for a significant minority of cancer diagnoses and provide important opportunities for high-impact clinical intervention (in probands and their relatives) through preventative strategies in unaffected individuals (e.g., surveillance scans, prophylactic surgery, and chemoprevention) and personalized therapies in those with cancer. Constitutional genetic variants in cancer-predisposition genes (CPGs) can predispose to a wide spectrum of tumors and levels of risk, although individual CPGs are usually associated with specific tumor types.[1]

Traditionally, genetic testing for inherited cancer syndromes has been performed on single or several CPGs selected according to the tumor phenotype in the individual or family. Latterly, next-generation sequencing (NGS) has transformed genetic diagnostics by enabling the

[1]University of Cambridge Department of Medical Genetics, NIHR Cambridge Biomedical Research Centre, and Cancer Research UK Cambridge Centre, Cambridge Biomedical Campus, Cambridge CB2 0QQ, UK; [2]NIHR BioResource, Cambridge University Hospitals, Cambridge Biomedical Campus, Cambridge CB2 0QQ, UK; [3]Department of Haematology, University of Cambridge, NHS Blood and Transplant Centre, Cambridge Biomedical Campus, Cambridge CB2 0PT, UK; [4]Department of Clinical Genetics, Princess Anne Hospital, Southampton SO16 5YA, UK; [5]North East Thames Regional Genetics Service, Great Ormond Street Hospital for Children, London WC1N 3JH, UK; [6]Yorkshire Regional Genetics Service, Chapel Allerton Hospital, Leeds LS7 4SA, UK; [7]Department of Clinical Genetics, University Hospitals of Leicester, Leicester Royal Infirmary, Leicester LE1 5WW, UK; [8]Peninsula Clinical Genetics, Royal Devon & Exeter Hospital, Exeter EX1 2ED, UK; [9]East Anglian Medical Genetics Service, Cambridge Biomedical Campus, Cambridge CB2 0QQ, UK; [10]West Midlands Regional Genetics Service and Birmingham Health Partners, Birmingham Women's and Children's Hospitals NHS Foundation Trust, Birmingham B15 2TG, UK; [11]Manchester Centre for Genomic Medicine, Division of Evolution and Genomic Sciences, University of Manchester, Manchester Academic Health Science Centre, St. Mary's Hospital, Manchester M13 9WL, UK; [12]Molecular Diagnostics Laboratory, National Centre of Scientific Research "Demokritos," Athens, Greece; [13]Department of Clinical Genetics, Liverpool Women's Hospital, Liverpool L8 7SS, UK; [14]Department of Clinical Genetics, St. George's Hospital, London SW17 0QT, UK; [15]Northern Genetics Service, Newcastle upon Tyne Hospitals, International Centre for Life, Newcastle upon Tyne NE1 3BZ, UK; [16]Department of Clinical Genetics, Guy's and St. Thomas' Hospital, London SE1 9RT, UK; [17]Division of Breast Surgery, University of Hong Kong, Pokfulam, Hong Kong; [18]Hong Kong Hereditary Breast Cancer Family Registry, Shau Kei Wan, Hong Kong; [19]Hong Kong Sanatorium and Hospital, Happy Valley, Hong Kong; [20]Clinical Genetics Institute, Kaplan Medical Center, Rehovot 76100, Israel; [21]Hebrew University and Hadassah Medical Center, Jerusalem, Israel; [22]Department of Clinical Genetics, Nottingham University Hospitals, City Hospital, Nottingham NG5 1PB, UK; [23]Department of Clinical Genetics, Aarhus University Hospital, Aarhus 8200, Denmark
*Correspondence: erm1000@medschl.cam.ac.uk
https://doi.org/10.1016/j.ajhg.2018.04.013.
© 2018 The Authors. This is an open access article under the CC BY license (http://creativecommons.org/licenses/by/4.0/).

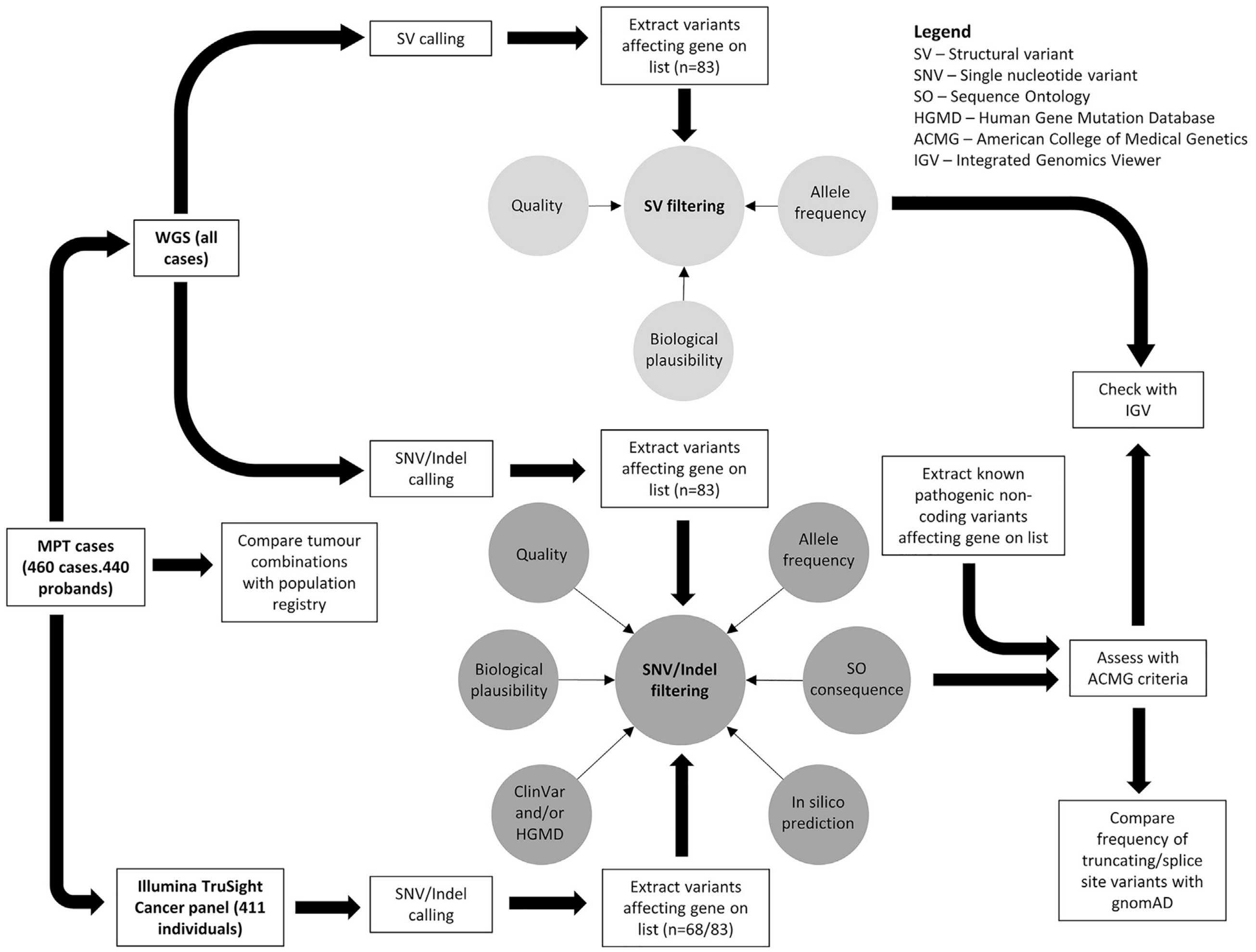

Figure 1. Study Design
Abbreviations are as follows: SV, structural variant; SNV, single-nucleotide variant; SO, Sequence Ontology; HGMD, Human Gene Mutation Database; ACMG, American College of Medical Genetics; and IGV, Integrated Genomics Viewer.

cost-effective analysis of large numbers of candidate genes. To date, the major factors prompting investigation for germline CPG variants have been family history and features of specific familial cancer syndromes. In addition, early age at cancer diagnosis and the occurrence of multiple primary tumors (MPTs) in the same individual are well recognized indicators of genetic susceptibility.[2,3] MPTs occur at appreciable frequency and are becoming more common with aging populations and increasing cancer survivorship.[4] Aside from genetic factors, non-genetic causes of MPT include environmental exposures relevant to multiple tumor types and carcinogenic cancer treatment.

Clinical NGS assays for possible inherited cancer predisposition generally take the form of single-gene or multigene panels of CPGs, but genome-wide analysis through whole-exome sequencing (WES) or whole-genome sequencing (WGS) is also possible. Although more expensive than WES, WGS should provide the most comprehensive analysis because it (1) can effectively interrogate all coding and non-coding areas of the genome, (2) provides more uniform read coverage than WES, particularly in areas where target enrichment and capture are difficult,[5,6] and (3) is able to detect a wide range of structural variations, such as deletions, translocations, and inversions.[7] However, WGS is still in its infancy as a clinical diagnostic tool, and few assessments of its application in hereditary cancer have appeared in the literature. In this study, we applied WGS to a large heterogeneous pre-assessed MPT cohort (460 individuals from 440 families) to investigate the potential role of comprehensive CPG analysis in this group.

Material and Methods

The study design is summarized in Figure 1.

Participants

460 participants from 440 families were recruited through clinical genetics services in the UK (442 individuals), Greece (nine individuals), Hong Kong (three individuals), the US (three individuals), Israel (two individuals), and Ireland (one individual). In each family, there was a clinical suspicion of a cancer-predisposition syndrome, but routine genetic assessment and testing had not

Table 1. Gene List Used for Analysis (n = 83)

AIP (MIM: 605555)	*EGFR* (MIM: 131550)[a]	*NF1* (MIM: 613113)	*SDHB* (MIM: 185470)
ALK (MIM: 105590)[a]	*EPCAM* (MIM: 185535)	*NF2* (MIM: 607379)	*SDHC* (MIM: 602413)
APC (MIM: 611731)	*ERCC2* (MIM: 126340)[b]	*NTHL1* (MIM: 602656)[b]	*SDHD* (MIM: 602690)
ATM (MIM: 607585)	*ERCC3* (MIM: 133510)[b]	*PALB2* (MIM: 610355)	*SERPINA1* (MIM: 107400)[b]
AXIN2 (MIM: 604025)	*ERCC4* (MIM: 133520)[b]	*PDGFRA* (MIM: 173490)[a]	*SMAD4* (MIM: 600993)
BAP1 (MIM: 603089)	*ERCC5* (MIM: 133530)[b]	*PHOX2B* (MIM: 603851)	*SMARCA4* (MIM: 603254)
BMPR1A (MIM: 601299)	*EXT1* (MIM: 608177)	*PMS2* (MIM: 600259)	*SMARCB1* (MIM: 601607)
BRCA1 (MIM: 113705)	*EXT2* (MIM: 608210)	*POLD1* (MIM: 174761)	*SMARCE1* (MIM: 603111)
BRCA2 (MIM: 600185)	*FH* (MIM: 136850)	*POLE* (MIM: 174762)	*SRY* (MIM: 480000)
BRIP1 (MIM: 605882)	*FLCN* (MIM: 607273)	*POLH* (MIM: 603968)[b]	*STK11* (MIM: 602216)
CDC73 (MIM: 607393)	*GATA2* (MIM: 137295)	*PRKAR1A* (MIM: 188830)	*SUFU* (MIM: 607035)
CDH1 (MIM: 192090)	*HFE* (MIM: 613609)[b]	*PTCH1* (MIM: 601309)	*TGFBR1* (MIM: 190181)
CDK4 (MIM:123829)[a]	*HNF1A* (MIM: 142410)	*PTEN* (MIM: 601728)	*TMEM127* (MIM: 613403)
CDKN1B (MIM: 600778)	*KIT* (MIM: 164920)[a]	*RAD51C* (MIM: 602774)	*TP53* (MIM: 191170)
CDKN2A (MIM: 600160)	*MAX* (MIM: 154950)	*RAD51D* (MIM: 602954)	*TSC1* (MIM: 605284)
CDKN2B (MIM: 600431)	*MEN1* (MIM: 613733)	*RB1* (MIM: 614041)	*TSC2* (MIM: 191092)
CEBPA (MIM: 116897)	*MET* (MIM: 164860)[a]	*RET* (MIM: 164761)[a]	*VHL* (MIM: 608537)
CHEK2 (MIM: 604373)	*MLH1* (MIM: 120436)	*RHBDF2* (MIM: 614404)[a]	*WT1* (MIM: 607102)
CYLD (MIM: 605018)	*MSH2* (MIM: 609309)	*RUNX1* (MIM: 151385)	*XPA* (MIM: 611153)[b]
DDB2 (MIM: 600811)	*MSH6* (MIM: 600678)	*SDHA* (MIM: 600857)	*XPC* (MIM: 613208)[b]
DICER1 (MIM: 606241)	*MUTYH* (MIM: 604933)[b]	*SDHAF2* (MIM: 613019)	

[a]Considered to be proto-oncogenes.
[b]Considered to be associated with tumor predisposition in the homozygous or compound-heterozygous state only.

identified a germline molecular genetic diagnosis at the time of recruitment. 435 individuals had developed MPTs (defined here as at least two primaries by age 60 years or at least three by 70 years), and 25 had developed a single primary tumor and had a first-degree relative with MPTs. Tumors in the same tissue type and organ were considered separate primary tumors if, in the case of paired organs, they occurred bilaterally or if the medical record clearly denoted them as distinct. International Agency for Research on Cancer criteria for defining separate primaries were also used.[8] Tumor diagnoses in the series were labeled according to site and cell of origin (Table S1).

All participants gave written informed consent to participate in the NIHR BioResource Rare Diseases, Molecular Pathology of Human Genetic Disease (HumGenDis), and/or Investigating Hereditary Cancer Predisposition (IHCAP) studies. The NIHR BioResource projects were approved by research ethics committees in the UK and appropriate ethics authorities in non-UK enrollment centers. Ethical approval for HumGenDis and IHCAP was given by the South Birmingham and East of England Cambridgeshire and Hertfordshire research ethics committees, respectively.

WGS and Panel Sequencing

WGS was performed on samples from study participants as part of the NIHR BioResource Rare Diseases study.[5] Blood DNA samples were fragmented (mean size 450 bp) with the Covaris LE220 kit and further processed with an Illumina TruSeq DNA PCR-Free Library Prep Kit. Libraries were sequenced with an Illumina HiSeq 2500 sequencer with one library per two lanes. FASTQ files were generated by HiSeq Analysis Software v.2.0 (Illumina). Alignment (GRCh37) and variant calling (including structural variants [SVs])[9,10] was performed with Isaac (Illumina).

For 411 samples, the Illumina TruSight Cancer Panel (TCP) was also used (gene list in Table S2), and libraries were sequenced with an Illumina MiSeq. BCL files resulting from the sequencing were converted to FASTQ files with Illumina's bcl2fastq. FASTQ files were checked for coverage and other quality-control parameters with fastqc software. FASTQ files were aligned to the UCSC Genome Browser (hg19) with the Burrows-Wheeler Aligner (BWA-MEM) with default parameters and SAMtools for the generation of a binary compressed sequence alignment map (BAM) files.[11,12] Variants were called from BAM files with the Genome Analysis Toolkit Unified Genotyper algorithm.[13,14] All data were annotated with Variant Effect Predictor (VEP) v.87 on the basis of canonical transcripts.[15]

SNV and Indel Identification and Assessment

Variants were extracted from VCF files if they were within a gene specified in a comprehensive list of 83 CPGs (Table 1) and had a predicted Sequence Ontology (SO) consequence indicating a deleterious effect on protein function. The gene list used for analysis was initially composed of all genes listed in a 2014 review of CPGs[1] (n = 114; gene list in Table S3) and/or those sequenced

by the Illumina TCP (n = 94). Two additional more recently described CPGs, namely *NTHL1* (MIM: 602656)[16] and *CDKN2B* (MIM: 600431),[17] were also included (Table S3). We subsequently reviewed and filtered the genes to produce a list that would be applicable to referrals to clinical cancer genetic services. Genes were included if deleterious variants affecting them were associated with adult-onset tumors and if neoplastic lesions were likely to be a primary presenting feature. For example, *SOS1* was not included because although Noonan syndrome is associated with increased neoplasia risk, other features of the condition are likely to prompt initial referral.

In order to identify clinically relevant variants, we subjected the resulting data to a range of filters (Figure S1). First, variants were removed if they failed to satisfy the quality criteria of a genotype quality (GQ) $\geq$ 30 (a Phred-scaled probability that the called genotype is incorrect), read depth (DP) $\geq$ 10 (at least ten reads covering the variant base[s]), variant allele fraction (VAF) $\geq$ 33%, and filter PASS (quality criteria applied by the Isaac variant caller in the NIHR BioResource Rare Disease Project). Second, variants were excluded if they had an allele frequency above 0.01 in either the Exome Aggregation Consortium (ExAC) Browser[18] (all populations) or the 1000 Genomes Project[19] (all populations). Third, variants were retained for further review if the predicted consequence was among a list of SO terms indicating protein truncation, if there was evidence of pathogenicity in ClinVar[20] (at least two-star evidence of pathogenic or likely pathogenic [P/LP] effect corresponding to multiple submissions with no conflicts as to the assertion of clinical significance), or if the variant was assigned a disease mutation (DM) status in the Human Gene Mutation Database (HGMD).[21] In order to consider a subset of non-truncating variants that are predicted to be pathogenic by *in silico* tools but do not appear in public databases, we also retained variants exceeding a Phred-scaled CADD[22] score threshold of 34 for further review. CADD was selected for this purpose given that it incorporates a range of tools and consequently a number of lines of evidence. The threshold was chosen as the median of scores assigned to other variants (affecting any gene) deemed pathogenic according to the criteria described below. Therefore, as a second variant filtering process, variants were identified for retention solely on the basis of CADD scores after variants retained for other reasons were assessed.

In the strategy described above, significant variants that are located in non-coding regions, such as introns, and affect genes in the gene list would not be extracted from the original VCF files because their SO consequence would not be in said list. Therefore, we used ClinVar to compile a list of known pathogenic variants falling outside of exons or splice sites and filtered VCFs on the basis of their genomic positions in a separate interrogation. Variants were incorporated in the list if they occurred in or near a gene in the list, were classified as near gene, non-coding RNA or untranslated region, and had at least two-star evidence of a P/LP effect. This process produced only three known pathogenic variants to search for in the WGS data. Distant non-coding variants affecting gene function (e.g., enhancers) were not considered in the current study.

Retained variants were subsequently excluded if their putative pathogenicity could be refuted by one of the following criteria: (1) a predicted protein-truncating variant for which there was at least two-star evidence of a benign or uncertain effect in ClinVar; (2) a predicted protein-truncating variant in a proto-oncogene in a list compiled on the basis of a literature review[1] (constitutional cancer-predisposing variants in proto-oncogenes are associated with gain-of-function variants, so truncation of the protein product is unlikely to increase tumor risk), (3) a predicted protein-truncating variant affecting <5% of the canonical transcript (according to the LOFTEE VEP plugin), (4) a variant affecting a gene associated with only recessive tumor predisposition (as defined by a literature review[1,16,23]) unless an individual appeared to harbor two filtered variants in the same gene, and (5) a variant with HGMD DM status or that exceeded the CADD score threshold and had at least two-star evidence of a benign or uncertain clinical effect or one-star evidence if there were multiple submissions without a P/LP assertion.

We used the Integrated Genomics Viewer (IGV)[24] to review variants that had passed filters to check for issues such as adjacent variants affecting the predicted consequence or variants being located at the end of sequencing reads. Pathogenicity was then assessed according to the American College of Medical Genetics (ACMG) criteria (Table S4),[25] which provide a framework for compiling multiple weighted lines of evidence. Additionally, for each variant, it was noted whether the corresponding individual had previously been diagnosed with a tumor typically associated with pathogenic variants in that gene (according to Rahman,[1] the Familial Cancer Database,[23] or the original paper reporting the gene as a CPG). Validation of P/LP variants was carried out with data from the TCP or Sanger sequencing according to standard protocols if TCP data were unavailable. Primer sequences are available on request.

SV Identification and Assessment

Structural variant (SV) calls that were predicted to affect a gene on the gene list (n = 83) were filtered and assessed according to the quality of the call, rarity of the variant, and biological plausibility of tumor predisposition caused by the variant (Figure S2). We initially filtered SVs called by Canvas and/or Manta to retain those that were predicted to affect at least one exon, occurred at a frequency of less than 1% across all NIHR BioResource Rare Disease samples (n = 9,110), and fulfilled minimum quality criteria (GQ $\geq$ 30 for Manta, QUAL $\geq$ 30 for Canvas). Remaining variants were regarded as potentially pathogenic if they affected a gene associated with tumor predisposition in the heterozygous state (unless there was evidence of homozygosity or compound heterozygosity) and fell into one of the following categories: (1) copy-number loss of coding regions of a tumor-suppressor gene, (2) copy-number gain of coding regions of a proto-oncogene, and (3) any SV type with a predicted breakpoint disrupting the gene. Subsequently, these SV calls were reviewed with IGV and excluded if they occurred in a copy-number variation map of the human genome[26] (hg19 stringent). The occurrence of tumors associated with disruption of particular genes in individuals harboring suspected SVs was noted in the same manner as for single-nucleotide variants (SNVs) and indels. BAM files corresponding to all suspected deleterious calls were reviewed in IGV. All SVs were confirmed with Sanger sequencing according to standard protocols. Inversions, translocations, and tandem duplications were confirmed by sequencing across breakpoints, whereas deletions were confirmed by fragment size resulting from long-range PCR if sequencing across the breakpoint was not possible. Primer sequences are available on request.

Comparison of MPT Series with Other Datasets

To consider how the tumor combinations in our series differed from those in the general population, we compared combination frequencies in our MPT data with a previously analyzed dataset from the East Anglia Cancer Registry (2009–2014; population size ~5.5 million). Registry data recorded individuals with two

Table 2. Most Frequent Tumors and Tumor Combinations in the Series

Tumor Category	Count	Percentage (%)
**>5% Total (n = 1,143)**		
Breast	281	24.6
Colorectal	113	9.9
Kidney	83	7.3
NMSC	67	5.9
Ovary	58	5.1
**>1% Total (n = 883)**		
Breast-colorectal	51	5.8
Breast-NMSC	35	4.0
Breast-ovary	34	3.9
Breast-endometrium	33	3.7
Breast-hem lymphoid	26	2.9
Breast-melanoma	24	2.7
Breast-thyroid	23	2.6
Endometrium-ovary	19	2.2
Breast-kidney	18	2.0
Colorectal-NMSC	14	1.6
Breast-lung	12	1.4
NMSC-hem lymphoid	11	1.2
Breast-soft tissue sarcoma	10	1.1
Colorectal-endometrium	9	1.0
Kidney-pituitary	9	1.0
Kidney-thyroid	9	1.0
Melanoma-NMSC	9	1.0

The following abbreviations are used: hem lymphoid, hematological lymphoid; and NMSC, non-melanoma skin cancer (including basal cell carcinoma and squamous cell carcinoma).

cancer (or central nervous system [CNS] tumor) diagnoses before the age of 60 years and only included tumors occurring before that age. Consequently, only combinations in MPT data of two malignant (or CNS) tumors occurring before 60 years of age were considered for this comparison.

To compare detection rates of loss-of-function variants in our cohort with a large-scale WGS dataset unselected for neoplastic phenotypes, we interrogated gnomAD[18] (data downloaded in February 2018) for variants occurring in the same set of 83 genes. Only truncating or splice-site variants were considered for comparison purposes because these are less likely to be false positives and made up 52/63 (82.5%) (see Results) of the P/LP variants in our cohort. Variants extracted from gnomAD were filtered and assessed in the same manner as those occurring in the MPT cohort. The frequency of variants assessed as P/LP was also calculated for males and females, and the sex distribution of individuals in the gnomAD dataset (55.3% male and 44.6% female) was estimated with mean allele count across all positions in the gnomAD VCF file of chromosomes 1–22. In order to estimate gnomAD P/LP variant frequency as though the sex distribution was equivalent to that in the MPT series (23% male and 77% female), we applied the sex-specific frequency to the estimated total number of gnomAD females (n = 6,929) and a reduced number of males (n = 2,064) that would achieve the desired proportion. We then summed the respective allele-frequency estimates to provide a figure for comparison with the MPT series.

Calculation of Coverage

For BAM files from WGS and TCP data, coverage statistics for regions of interest were generated with SAMtools depth.[12] A BED file compiled with Ensembl BioMart[27] to represent translated exonic regions and splice sites of genes in the gene list was utilized for this purpose.

Statistical Analysis

All statistical tests were performed with R v.3.4.3.[28] Pearson's χ^2 tests and Student's t tests were performed with the chisq.test and t.test functions, respectively.

Results

Clinical Characteristics and MPT Combinations

460 individuals (106 [23%] males and 354 [77%] females) in 440 families had been diagnosed with 1,143 primary tumors distributed among 87 categories according to site and cell of origin. The most frequent tumor types are illustrated in Table 2 (comprehensive lists are provided in Tables S1 and S5). Representing 24.6% of the total, breast cancer was the most frequent tumor, and colorectal cancer was the second (9.9%). Prior genetic testing is described in Table S6, and reasons for non-detection of the relevant variant are illustrated in Figure 2.

The occurrence of any two discordant primary tumors in the same individual was considered a tumor combination, and a total of 883 combinations and 327 combination types were observed (individuals with three or more discordant tumors had multiple combinations). 206 (63%) combination types occurred once, and 53 (16.2%) occurred twice. The 68 (20.8%) combination types occurring three or more times are illustrated in Figure 3. The most frequent combination type was breast and colorectal cancer, which represented 5.8% of the total combinations. All combination types making up $\geq$1% of the total are shown in Table 2.

To compare the distributions of tumor combinations in our MPT study cohort with a population-based dataset, we compared 313 MPT cohort individuals comprising 523 combinations with 471 individuals comprising 574 combinations in the East Anglia Cancer Registry data (Table S7). There was a significant difference (χ^2 p value < 0.05) in the frequency of tumor combinations in 6/12 combination types that individually represented >1% of the total MPT cohort. Breast cancer in combination with ovarian, thyroid, lymphoid hematological, or kidney cancer was overrepresented in the MPT cohort, whereas breast cancer in combination with non-melanoma skin was underrepresented.

Information regarding previous genetic testing was available for 405/440 (92%) probands. No molecular

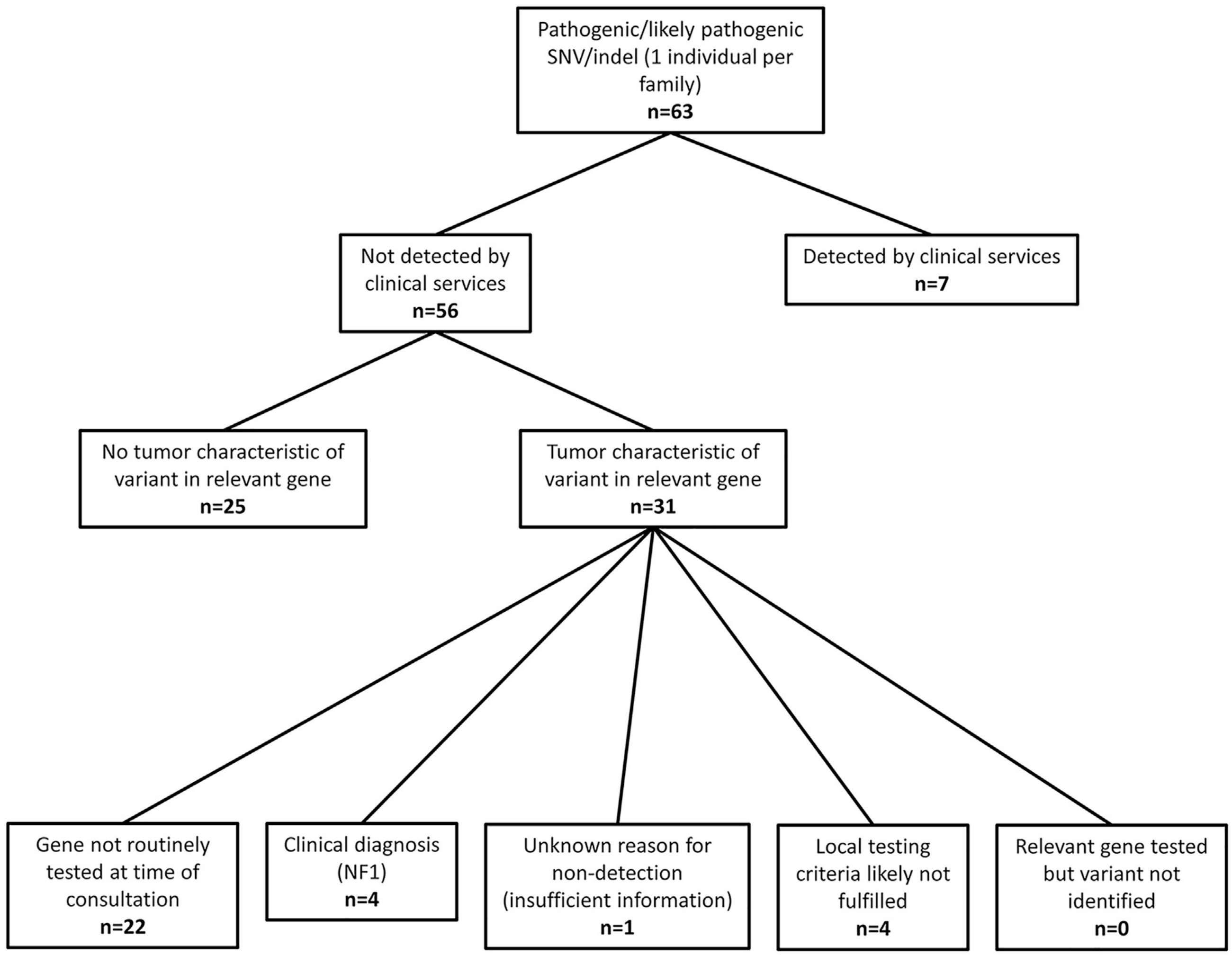

Figure 2. Molecular Investigations Initiated by Clinical Services with Inferred Reasons for Non-detection of Variants

investigations had been performed in 91 (20.7%). 159 (36.1%) had undergone BRCA1 and BRCA2 testing, 87 (19.8%) had been assessed for Lynch syndrome (where microsatellite instability [MSI] and/or immunohistochemistry [IHC] analysis is considered an assessment), and 159 (20.7%) had had another germline genetic test. The mean number of genes analyzed (where MSI or IHC is considered an analysis of four Lynch syndrome genes) was four. Samples from 79 (18%) probands had undergone sequencing with a multi-gene panel assay, and the mean number of genes analyzed with these assays was 13.8.

Genetic Findings

SNVs and Indels

Variant filters applied to annotated VCF files produced 89 unique variants in 119 individuals for further ACMG-guideline-based assessment. Of these, 22 (42 occurrences) could be classified as pathogenic, 23 (24 occurrences) could be classified as likely pathogenic, 24 (27 occurrences) could be classified as a variant of uncertain significance (VUS), and 20 (26 occurrences) could be classified as likely benign. Six occurrences of P/LP variants occurred in two members of the same family, and only three of these contributed to the detection rates quoted below. No pathogenic non-coding variants were identified.

Overall, 63 variants in 17 genes in 61 (13.9%) probands were assessed as P/LP (summary in Table 3; full description with phenotype and previous testing in Table S6). Most were nonsense or frameshift variants. Individuals with variants in moderate-risk CPGs *CHEK2* (MIM: 604373; n = 14) and *ATM* (MIM: 607585; n = 10) were the most frequent; one homozygote was detected for *CHEK2*: c.1100delC (p.Thr367Metfs) (Ensembl: ENST00000328354; GenBank: NM_007194.3]; annotated in our data as c.1229delC [p.Thr410fs] [Ensembl: ENST00000382580; GenBank: NM_001005735.1]). Individuals with variants in *BRCA2* (MIM: 600185; n = 6), *PALB2* (MIM: 610355; n = 6), *FH* (MIM: 136850; n = 5), *NF1* (MIM: 613113; n = 4), *NTHL1* (MIM: 602656; homozygous, n = 3), *MAX* (MIM: 154950; n = 2), *PTEN* (MIM: 601728; n = 2), *SDHB* (MIM: 185470; n = 2), *BMPR1A* (MIM: 601299; n = 1), *BRCA1* (MIM: 113705; n = 1), *CDKN1B* (MIM: 600778; n = 1), *EXT2* (MIM: 608210; n = 1), *MLH1* (MIM: 120436; n = 1), MSH2

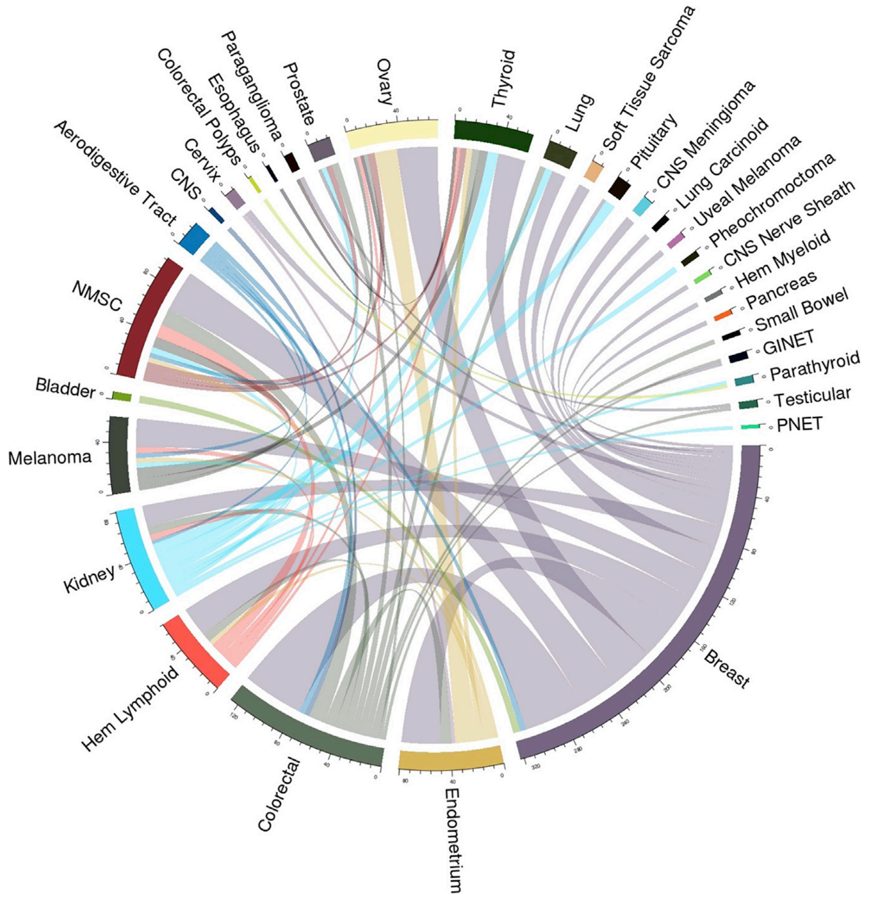

Figure 3. Most Frequent Tumor Combination Types
Combination types occurring fewer than three times are not included. Abbreviations are as follows: pheo, pheochromocytoma; GI NET, gastrointestinal neuroendocrine tumor; hem myeloid, hematological myeloid; PNET, pancreatic neuroendocrine tumor; hem lymphoid, hematological lymphoid; and NMSC, non-melanoma skin cancer (including basal cell carcinoma and squamous cell carcinoma).

(MIM: 609309; n = 1), and *PMS2* (MIM: 600259; n = 1) were also noted.

The 61 P/LP SNV and indels detected by WGS were confirmed by a second analysis (TCP for 51 variants and Sanger sequencing for ten variants).

Pre-testing information was available for 57/63 P/LP variants, 41/57 (71.9%) of which occurred in an individual who had at least one previous genetic test and 7/57 (12.3%) of which were eventually detected by clinical services. No P/LP variants were observed in genes that had previously been tested in a sample from the relevant individual by diagnostic services (Figure 2). The mean number of genes tested in those with a P/LP variant was 5.3, which was not significantly different from that in probands without such variants detected (Student's t test $p = 0.396$).

Of the 61 probands identified with a P/LP variant, 36 (59%; 8.2% of all probands) had previously been diagnosed with a tumor typically associated with the relevant CPG. A further eight (1.8%) probands were found to harbor a VUS and had been diagnosed with an associated tumor.

Two probands harbored two P/LP variants in multiple CPGs. One individual with colorectal adenocarcinoma at age 50 years and breast cancer at 57 years carried a *PMS2* frameshift variant (c.741−742insTGAAG [p.Pro247_Ser248fs] [Ensembl: ENST00000265849; GenBank: NM_000535.6]) and a *BMPR1A* nonsense variant (c.730C>T [p.Arg244*] [Ensembl: ENST00000372037; GenBank: NM_004329.2]). Immunohistochemistry of the bowel tumor showed loss of PMS2; MSI was also demonstrated, leading to diagnostic sequencing of *PMS2*, although there was no family history of neoplasia other than an ovarian cancer in a second-degree relative after age 70 years. The proband had previously undergone surveillance colonoscopy for inflammatory bowel disease, resulting in the identification of a number of polyps; however, there was no evidence from histology reports that these were juvenile polyps. Additionally, an individual with bilateral pheochromocytoma at ages 16 and 35 years and no reported family history of neoplasia was identified with variants in *FH* (c.521C>G [p.Pro174Arg] [Ensembl: ENST00000366560; GenBank: NM_000143.3]) and *MAX* (c.1A>G [p.Met1Val] [Ensembl: ENST00000358664; GenBank: NM_002382.4]).[29] The latter variant is predicted to abolish the *MAX* initiation codon, and previous analysis of tumor tissue from an individual carrying it demonstrated loss of the wild-type allele and a lack of full-length *MAX* protein product.[30]

Coverage and Comparison with Panel

Mean depth in WGS data of coding bases in the 83 genes analyzed was 35× (SD = 7.5), and 100% were covered at ≥10×. Coverage was also considered for 68 of the genes also sequenced by the TCP. In WGS data, 100% of target bases were covered at ≥10×, and the mean depth was 35.3 (SD = 7.4). Coverage analysis pertaining to those 68 genes from the 411 (89.3%) participants also undergoing sequencing with the TCP showed 99.1% target bases at ≥10× and a mean depth of 807.3 (SD = 793.2).

A comparison of the variant detection was performed on the basis of the 105 ACMG-assessed SNVs and indels that were detected by WGS and were within a gene sequenced by the TCP. 99/105 variants were called from TCP data with quality indicators sufficient to pass filters used for the WGS data. Five undetected variants—including one P/LP *PMS2* variant (c.741−742insTGAAG

Table 3. Summary of Filtered SNVs and Indels Deemed Pathogenic or Likely Pathogenic by ACMG Assessment

Gene	RefSeq mRNA ID	No. of Occurrences	No. of Individuals with Associated Tumor	Variant Description	Consequence
ATM	GenBank: NM_000051	1	1	c.193C>T (p.Gln65*)	stop gain
ATM	GenBank: NM_000051	1	1	c.5623C>T (p.Arg1875*)	stop gain
ATM	GenBank: NM_000051	1	0	c.6583+1G>A	splice site (donor)
ATM	GenBank: NM_000051	1	0	c.6866-6867insT (p.Ser2289Serfs)	frameshift
ATM	GenBank: NM_000051	1	1	c.748C>T (p.Arg250*)	stop gain
ATM	GenBank: NM_000051	1	1	c.8147T>C (p.Val2716Ala)	missense
ATM	GenBank: NM_000051	1	0	c.8405delA (p.Gln2802fs)	frameshift
ATM	GenBank: NM_000051	1	0	c.5821G>C (p.Val1941Leu)	missense
ATM	GenBank: NM_000051	1	0	c.8122G>A (p.Asp2708Asn)	missense
ATM	GenBank: NM_000051	1	1	c.7775C>G (p.Ser2592Cys)	missense
BMPR1A[a]	GenBank: NM_004329	1	1	c.730C>T (p.Arg244*)	stop gain
BRCA1	GenBank: NM_007300	1	1	c.1961−1962insA (p.Lys654fs)	frameshift
BRCA2	GenBank: NM_000059	1	0	c.4525C>T (p.Gln1509*)	stop gain
BRCA2	GenBank: NM_000059	1	1	c.5682C>G (p.Tyr1894*)	stop gain
BRCA2	GenBank: NM_000059	1	0	c.6275−6276delTT (p.Leu2092fs)	frameshift
BRCA2	GenBank: NM_000059	1	1	c.6402−6406delTAACT (p.Asn2135Leufs)	frameshift
BRCA2	GenBank: NM_000059	1	0	c.6535−6536insA (p.Val2179fs)	frameshift
BRCA2	GenBank: NM_000059	1	1	c.1805−1806insA (p.Gly602fs)	frameshift
CDKN1B	GenBank: NM_004064	1	0	c.148−149delAG (p.Arg50fs)	frameshift
CHEK2	GenBank: NM_001005735	3	1	c.1392delT (p.Leu464fs)	frameshift
CHEK2	GenBank: NM_001005735	10	6	c.1229delC (p.Thr410fs)	frameshift
CHEK2	GenBank: NM_001005735	1	1	c.1051+1C>T	splice site (donor)
CHEK2	GenBank: NM_001005735	1	0	c.784delG (p.Glu262fs)	frameshift
CHEK2	GenBank: NM_001005735	1	1	c.562C>T (p.Arg188Trp)	missense
EXT2	GenBank: NM_000401	1	0	c.613C>T (p.Gln205*)	stop gain
FH	GenBank: NM_000143	3	0	c.1433−1434insAAA (p.Lys477_Asn478insLys)	in-frame insertion
FH	GenBank: NM_000143	1	1	c.320A>C (p.Asn107Thr)	missense
FH[b]	GenBank: NM_000143	1	0	c.521C>G (p.Pro174Arg)	missense
MAX	GenBank: NM_002382	1	1	c.289C>T (p.Gln97*)	stop gain
MAX[b]	GenBank: NM_002382	1	1	c.1A>G (p.Met1Val)	start loss
MLH1	GenBank: NM_000249, NM_001258273	1	1	c.1884−1G>A	splice site (acceptor)
MSH2	GenBank: NM_000251	1	0	c.1452−1455insAATG (p.Leu484-Met485fs)	frameshift
NF1	GenBank: NM_001042492	1	1	c.1541−1542delAG (p.Gln514fs)	frameshift
NF1	GenBank: NM_001042492	1	1	c.4620delA (p.Ala1540fs)	frameshift
NF1	GenBank: NM_001042492	1	1	c.5831delT (p.Leu1944fs)	frameshift
NF1	GenBank: NM_001042492	1	1	c.7768-7769insA (p.His2590fs)	frameshift
NTHL1[c]	GenBank: NM_002528	3	3	c.268C>T (p.Gln90*)	stop gain
PALB2	GenBank: NM_024675	4	3	c.3113G>A (p.Trp1038*)	stop gain
PALB2	GenBank: NM_024675	1	1	c.3116delA (p.Asn1039fs)	frameshift

(*Continued on next page*)

Table 3. *Continued*

Gene	RefSeq mRNA ID	No. of Occurrences	No. of Individuals with Associated Tumor	Variant Description	Consequence
PALB2	GenBank: NM_024675	1	1	c.62T>G (p.Leu21*)	stop gain
PMS2[a]	GenBank: NM_000535	1	1	c.741−742insTGAAG (p.Pro247_S248fs)	frameshift
PTEN	GenBank: NM_000314	1	1	c.1003C>T (p.Arg335*)	stop gain
PTEN	GenBank: NM_000314	1	1	c.697C>T (p.Arg233*)	stop gain
SDHB	GenBank: NM_003000	1	1	c.223+1C>A	splice site (donor)
SDHB	GenBank: NM_003000	1	1	c.689G>A (p.Arg230His)	missense

This list incorporates one individual per family. See Table S6 for more comprehensive description.
[a]Occurring in the same individual.
[b]Occurring in the same individual.
[c]Homozygous.

[p.Pro247_Ser248fs] [Ensembl: ENST00000265849; GenBank: NM_000535.6]), where 58/202 (20.6%) reads contained the insertion—were indels for which IGV review showed a VAF below the threshold for filtering. One undetected variant in *TMEM127* (MIM: 613403) (c.665C>T [p.Ala222Val] [Ensembl: ENST00000258439; GenBank: NM_017849.3]) was covered by only two reads.

The filtering and assessment process applied to WGS data was also used for variants called from TCP data generated from the same 411 individuals. 108/110 TCP variants that passed filters and went forward for ACMG assessment were also called from WGS data, meaning that two variants (assessed as pathogenic) were not detected by WGS. This was because the VAF was marginally below the filtering threshold of 33% for *ATM* (c.2426C>A [p.Ser809*] [Ensembl: ENST00000278616; GenBank: NM_000051]) (7/22 [32%] reads) and *MAX* (c.97C>T [p.Arg33*] [Ensembl: ENST00000358664; GenBank: NM_002382]) (9/29 [31%] reads).

Comparison of MPT WGS SNV and Indel Detection with gnomAD Dataset

In our dataset, 52 truncating or splice-site variants were observed in 440 MPT probands, whereas 298 were observed in 8,992 gnomAD genomes; the latter is based on observed variant frequency estimates adjusted to reflect sex distribution of the MPT series (13.6% for the MPT dataset versus 3.3% for the gnomAD dataset; $\chi^2 = 84.903$, $p = < 0.0001$). 41 truncating or splice-site CPG variants occurred in a proband with at least one tumor type uncharacteristic of the relevant CPG, and the frequency of such variants in these individuals was also compared with that in gnomAD. This was significantly higher in the MPT probands with uncharacteristic tumors than in gnomAD (41/440 [9.3%] versus 298/8,992 [3.3%]; $\chi^2 = 43.642$; $p \leq 0.0001$).

SVs

SV analysis revealed six potentially pathogenic variants in 440 (1.4%) probands (Table 4), two of whom had previously been diagnosed with tumors typically associated with variants in the relevant gene. An additional two had no associated tumor but a family history of such tumors in a first-degree relative (colorectal cancer at age 56 years for the individual with a *SMAD4* translocation and renal cell carcinoma at age 69 for the individual with the *TSC1* duplication). One individual with an inversion of *PTEN* exon 7 had been diagnosed with breast cancer at age 45 years and had a strong family history of this tumor, which had occurred in her sister (age 57 years), mother (age 57 years), and maternal cousin (age 49 years). The proband's sister had also been diagnosed with a borderline ovarian mucinous tumor and nasal basal cell carcinoma at 46 and 57 years of age, respectively, but WGS did not detect the *PTEN* inversion in her sample. Another individual had previously been investigated with germline *FH* sequencing after the diagnosis of multiple cutaneous leiomyomas and a family history of a first-degree relative undergoing a hysterectomy for uterine leiomyomas. SV analysis revealed a whole-gene deletion of *FH*.

Combined Variant Detection Rate

After combining SVs passing our filters and ACMG-assessed P/LP SNVs and indels, we observed a P/LP variant in 67 (15.2%) probands tested. 38 probands (8.6% of total) had such a variant and a typically associated tumor. There was no significant difference in P/LP detection rate between probands diagnosed with a rare tumor and those who hadn't been (27/136 [19.8%] versus 40/304 [13.1%]; $\chi^2 = 3.2628$; $p = 0.07087$). Of the 55/67 probands for whom a family history was available, there was no cancer diagnosis in a first-degree relative younger than 50 years in 23 individuals (61.8%) and younger than 60 years in 34 individuals (61.8%).

Limited numbers of family members participated in the study, preventing large-scale segregation analysis. Of the 69 P/LP variants (including SVs) of interest detected in probands, the relevant locus was sequenced in a family member on seven occasions. The relevant variant was detected in four of seven family members, two of whom had been diagnosed with a typically

Table 4. Structural Variants Passing Filtering Steps

Gene	Chr	Predicted Start	Predicted End	Algorithm(s)	Predicted Consequence after IGV Review	Phenotype (Age at Diagnosis)	Genes Tested by Clinical Services	Year Consulted
FLCN	17	1,7136,696 (Manta), 1,7137,867 (Canvas)	17,134,310 (Manta), 17,134,474 (Canvas)	Canvas and Manta	deletion of exon 2	breast (46 years) and pulmonary lymphangioleiomyomatosis (47 years)	information unavailable	unknown
PTEN	10	89,719,837	89,713,996	Manta	inversion of exon 7	breast (45 years)[a]	*BRCA1* and *BRCA2* (single gene)	unknown
SMAD4	9 and 18	chr9: 127,732,713	chr18: 48,556,624	Manta	translocation with breakpoint within untranslated part of exon 1	CNS (42 years) and colorectal (56 years) in mother	*PMS2*, *TP53*, and *MLH1* (single gene)	2011
TSC1	9	135,807,261	135,803,187	Manta	duplication of exon 3	testicular (47 years), prostate (64 years), and lung (70 years)	*BRCA1* and *BRCA2* (single-gene Ashkenazi common mutations)	2016
TSC2	16	2,119,769	1,566,500	Manta	inversion with breakpoint in introns 16 and 17	small bowel (42 years) and colorectal (43 years)	*MSH6* (single gene; IHC also revealed MSH6 loss)	2012
FH	1	242,310,908	237,244,834	Canvas	full-gene deletion	multiple cutaneous leiomyomata (<55 years)[a]	*FH* (single gene)	2014

The list incorporates one individual per family. All structural variants are heterozygous. The following abbreviations are used: Chr, chromosome; CNS, central nervous system; IHC, immunohistochemistry.
[a]Tumor characteristically associated with pathogenic variant in the relevant gene.

associated tumor (breast cancer in *PALB2* and *BRCA1* variants).

Discussion

Variant Detection Rates in an MPT Series

We previously reported a retrospective series of MPT individuals (defined as having two primary tumors before 60 years of age) referred to a UK clinical genetics service without pre-assessment and observed that 20.7% (44/212) were found to have a molecular diagnosis upon routine targeted molecular genetic testing, including BRCA1 and BRCA2 testing, mismatch-repair gene analysis, or other single-gene testing (*APC* [MIM: 611731], *MUTYH* [MIM: 604933], *PTEN*, *TP53* [MIM: 191170], and *RB1* [MIM: 614041]).[31]

In the current study, we addressed whether comprehensive genetic analysis in pre-assessed individuals with MPTs might increase the diagnostic yield over routine targeted testing. Thus, we analyzed 460 MPT-affected individuals who had previously undergone routine genetic assessment and/or molecular testing (but without a molecular diagnosis) by using WGS for variants in 83 CPGs and identified a P/LP variant in 67/440 (15.2%) probands (incorporating SNVs, indels, and SVs), including those affected by moderate- and high-risk CPGs.

Because the MPT cohort reported here was mostly ascertained from UK genetics centers (and was similar to the previous retrospective cohort that did not have a known genetic cause), we estimate (by assuming that WGS would detect variants identified by routine targeted sequencing approaches) that comprehensive genetic analysis in a genetics-center-referred series of individuals with MPTs (and no prior genetic testing) would detect a P/LP variant in around a third of individuals (20.7% + 12.1% [estimated under the assumption of a diagnostic yield of 15.2% in the 79.3% of individuals without a variant in routine testing] = 32.8%). The estimated proportion of individuals with a P/LP variant and a typical tumor would be ~27.5% (20.7% [all of those with variants detected by targeted analysis had a typical tumor] + [79.3% × 8.6% = 6.8%]). Therefore, in individuals seen in a genetic clinic, the presence of MPTs (two tumors before 60 years of age or three before 70 years of age) could be taken as an indication for considering genetic testing.

The estimates for diagnostic yield are approximate and would be influenced by ascertainment processes but do suggest that a comprehensive testing for CPG variants significantly increases the detection of P/LP variants over the targeted testing that has been routinely employed in most genetics centers.

Most MPT-affected individuals (38/67 [56.7%] and 38/440 [8.6%] of all pre-assessed probands tested in the current study) with a P/LP variant had been diagnosed with a tumor type characteristically associated with

variants in the relevant CPG, findings that have the greatest clinical utility. In, addition, a further 8/440 (1.8%) had a VUS and a previous diagnosis of a characteristic tumor. Such VUSs might eventually be reclassified as LP variants with further investigations (e.g., tumor studies or functional analysis) or additional clinical information (e.g., segregation analysis). However, interpretation of segregation data should be cautious in cancer-predisposition syndromes because of incomplete penetrance and a higher probability of phenocopies. Tumor studies for loss of heterozygosity do not provide absolute confirmation or exclusion of pathogenicity, and together these considerations reinforce the importance of data-sharing initiatives such as ClinVar.[20]

A major influence on the number and pattern of variants detected in a study such as this is the tumor phenotypes occurring in the cohort, which in this case reflect both the population incidence and the patterns of referral for genetic assessment and investigation. Compared with MPT-affected individuals in cancer registries, our series is enriched with combinations such as breast-ovary (4.4% versus 1.9%) and breast-colorectal (5.5% versus 2.8%), most likely reflecting common cancers with a significant hereditary component and for which genetic testing has been routinely available for a number of years. Many of these cancers are sex specific, most likely contributing to the uneven sex distribution in this series. Some combination types making up >1% of MPT combinations, e.g., breast-thyroid (3.6% in MPT data), are not observed frequently (<1%) in the population-based cohort used here, which could be accounted for by referral prompted by suspicion of germline *PTEN* variants.

Breast cancer accounted for almost a quarter of tumors in our series, and most genes in which deleterious variants were detected are breast CPGs, many of which are not routinely tested in the UK. Pathogenic variants in *ATM* and *CHEK2* are associated with moderate risks,[32,33] and these genes had not been tested by the referring center in any of the individuals with P/LP variants. Six probands had pathogenic variants in *PALB2*, a gene initially thought to confer moderate risk[34] but subsequently reported to have a penetrance somewhere between that of moderate- and high-risk genes such as *BRCA1* and *BRCA2*.[35]

Genes can remain uninvestigated by clinicians not only because of uncertainty surrounding risks but also because of recency of discovery. A number of CPGs in which variants were identified, such as *MAX* and *FH*, have been relatively recently described as causing pheochromocytoma and paraganglioma. The appearance of these variants in this analysis most likely reflects a lack of availability of testing at the time of consultation and subsequent referral for inclusion in the study. Molecular genetic testing has been available for other genes such as *MLH1* and *PTEN* for a greater period of time, but some individuals appeared not to have fulfilled the clinical testing criteria applied in the referring center. *TP53* is a further well-established CPG that is associated with diverse and multiple cancers and has clear clinical testing criteria that are often not fulfilled. Despite this, no pathogenic variants were detected. Germline *TP53*-variant-related phenotypes (including rare and/or early-onset cancers) are more clearly identifiable clinically and are less likely to appear in cohorts such as ours without specific ascertainment for them. Consistent with this are mutation detection rates of ∼4% in individuals with earlier-onset (≤30 years) breast cancer[36] and ∼17% in MPT-affected individuals who were referred for germline *TP53* testing and who generally fulfilled criteria for that investigation, had tumors characteristic of Li Fraumeni syndrome, and had an average age at diagnosis (of a first primary tumor) before 30 years.[2]

Although we report the application of WGS to an adult MPT series, other studies have used agnostic NGS strategies in cohorts with single-site cancer. The detection rate of pathogenic variants in these analyses could be influenced by the assay used, the variant filtering and assessment applied, and the nature of the series in terms of both phenotype and ascertainment. The application of a 76-gene panel to ∼1,000 cancer-affected adults referred for germline genetic testing and ACMG-guideline-based assessment of the resulting variants showed a 17.5% rate,[37] whereas tumor-normal sequencing of a similarly sized series with advanced cancer from the same center (regardless of genetic testing referral) reported an equivalent figure of 12.6%.[38] The genes containing the most frequent pathogenic variants in both studies were similar to those in the current study (*BRCA1*, *BRCA2*, *CHEK2*, and *ATM*), but the detection rates were lower than our estimate of around a third of newly referred MPT-affected individuals, most likely reflecting a greater likelihood of a germline pathogenic variant in both genetics referrals and in MPT-affected individuals. Studies of WGS and/or WES applied to unselected pediatric cancer series have also shown pathogenic-variant detection rates close to 10% but a contrasting range of affected genes, suggesting that *TP53* and genes associated with embryonal tumors play a far greater role.[39–41]

Atypical Tumor-Variant Associations in MPT-Affected Individuals

In this study, we applied multi-gene testing in all affected individuals irrespective of the tumor types diagnosed. Strikingly, this resulted in the identification of a large number of probands (29/67 [43.2%]) who harbored a P/LP CPG variant but whose tumor phenotypes were not entirely typical for the relevant CPG. This situation has been frequently reported by other studies of extensive NGS testing of cancer cohorts[37,40,42] and represents a challenge for clinicians because the relevance of the variant to cancer risk in the consultand (including unaffected family members) is less clear. Specific atypical associations observed in this analysis were heterogeneous, and numbers were small, but some patterns were noted; for example, 5/16 (31.2%) carriers of *CHEK2* variants had been previously diagnosed with renal cell carcinoma

(RCC) (breast cancer occurred in 8/16 [50%]). An odds ratio of 2.1 for RCC has previously been observed in *CHEK2*-variant carriers but only in association with the c.470T>C (p.Ile157Thr) founder variant in a Polish population.[43] 2/6 (33.3%) carriers of *PALB2* variants had cutaneous melanoma before the age of 40 years, and 2/10 (20%) individuals with *ATM* variants had thyroid cancer before that age, but an analysis of 182 melanoma families demonstrated only one pathogenic *PALB2* variant,[44] and thyroid malignancies have not been reported at increased frequency in carriers of homozygous or heterozygous *ATM* variants.[1,45]

One potential interpretation of these atypical tumor phenotypes is that the tumor spectrum associated with some CPGs is wider than currently recognized given that, to date, testing of particular genes has been limited to specific phenotypes. For example, although *FH* variants were demonstrated to predispose to RCC in 2002, they were shown to predispose to pheochromocytoma and paraganglioma 12 years later.[46–48] We therefore suggest that further "agnostic" research testing of a comprehensive panel of CPGs in MPT-affected individuals could lead to the identification of novel associations between genes and tumor phenotypes. Our observation of a significantly higher rate of loss-of-function variants associated with non-characteristic tumors in our cohort than in the gnomAD dataset suggests that at least some variants identified in individuals with atypical phenotypes are relevant. We would, however, urge caution in automatically linking a pathogenic CPG variant to the observed tumor phenotype without further evidence, such as larger studies of variant carriers or tumor studies that demonstrate a variant's causative effect.

Another possibility is that tumors can occur coincidentally in the presence of a pathogenic constitutional CPG variant. Variants might be considered causative in some contexts or tissues (and would therefore be likely to pass our filtering and assessment) but potentially not in others. For example, an in-frame *FH* insertion (c.1433−1434insAAA [p.Lys477_Asn478insLys] [Ensembl: ENST00000366560; GenBank: NM_000143.3]) was identified in three individuals, none of whom had been diagnosed with typical hereditary leiomyoma or RCC tumors. This variant causes recessively inherited fumarate hydratase deficiency (MIM: 606812) and has been demonstrated to disrupt enzyme activity.[49] However, its significance to cancer predisposition in the heterozygous state is less well defined.

Unusual MPT-CPG associations can occur when an individual harbors variants in multiple CPGs, either because (at least) one of the variants remains unidentified through diagnostic testing or because an interactive effect exists between them. We have previously reviewed this phenomenon and described it as multiple inherited neoplasia alleles syndrome (MINAS),[50] and WGS identified two further examples in our cohort. In the case of *PMS2* and *BMPR1A* variants, the former appears to be penetrant on the basis of tumor studies, whereas the significance of the latter is unclear. Nevertheless, the identification of MINAS cases such as this provides clinicians the opportunity to obtain further evidence. For the individual with *FH* and *MAX* variants, it is easier to attribute the diagnosed pheochromocytomas to the truncating *MAX* variant, but evidence for the role of *FH* in this tumor type is accumulating, and this variant could have contributed to tumorigenesis.

Value of Germline WGS in the Analysis of MPTs

Although WGS could arguably offer the most sensitive and comprehensive strategy for detecting germline CPG variants, it is resource intensive in terms of sequencing, data storage, and analytical capacity. In this study, the conservative variant filtering and assessment and the small number of non-coding variants that were used for data interrogation reduced the post-sequencing burden of variants, but small changes to these processes would lead to significant increases with uncertain clinical utility. The approximate cost per sample of WGS as part of the NIHR BioResource Rare Disease project is $1,400, consistent with figures collated by the National Human Genome Research Institute in 2016 and higher than the $1,000 per exome derived from that survey.[51] The TCP in our department is currently charged at around $450 per sample. Justification of the extra costs compared with those of other NGS assays, such as panel tests or WES, requires the demonstration that WGS can increase the diagnostic rate over that of other approaches through enhanced detection of coding SNVs and indels, SV identification, or analysis of non-coding regions.

In our analysis, the TCP produced a higher mean depth but a slightly lower percentage of target bases covered at ≥10× than the equivalent regions in WGS data (99.1% versus 100%). WGS identified one *TMEM127* SNV (assessed as a VUS) that wasn't detected by the TCP because the relevant nucleotide was covered by only two reads. Five additional filtered variants from the WGS data weren't called from panel data, and one of them was assessed as likely pathogenic. This was because the VAF was marginally below the chosen threshold, an issue that also accounted for the calling of two pathogenic variants from TCP data but not from WGS. Non-detection of lower-VAF variants could be resolved through more sensitive bioinformatic filtering of data from either assay. 15 genes on our list of 83 were not targeted by the panel (but no significant variants were detected in them). This illustrates the broader scope of WGS, but our results do not suggest that WGS offers enhanced CPG SNV or indel detection at present.

WGS identified six SVs predicted to affect a gene of interest, and two of these occurred in an individual whose personal or family history included tumors consistent with variants in that gene. The medical record showed no evidence that the individual with the *PTEN* inversion exhibited other features of constitutional variants in this

gene, such as macrocephaly, as well as no record of an examination in a consultation where only *BRCA1* and *BRCA2* testing was anticipated. Although the numbers of potentially pertinent SVs are small, these aberrations are unlikely to be detected by panel or exome sequencing alone. Copy-number variation can be identified from the analysis of read counts in WES or panel data,[52] but most diagnostic laboratories rely on techniques such as multiplex probe ligation assays (MLPAs) to test individual genes. If MLPA analysis is applied to many genes, then the cost could make WGS more economical than WES or panel-based testing, but investigating this would require a detailed cost-benefit analysis. Furthermore, WGS can detect inversions and translocations that are not characterized by MLPA. A note of caution, however, arises from a deletion involving *BRCA2* exons 14–16; we were made aware of this deletion by the referring clinician, but it was not detected through our analyses.

Given the limited benefits of WGS over WES and panel analysis demonstrated in this study, a key advantage is the ability to prospectively or retrospectively interrogate regions that are not currently known to be clinically relevant. This includes novel CPGs (many of the P/LP variants in this analysis were detected because the gene or region was not available for testing at the time of consultation). WGS costs should therefore be considered in the context of possible future demand for re-investigation and the consequent resource burden required for this if the region of interest (including non-coding regions) is not sequenced in the first instance. Adequate systems for prioritizing and assessing the multitude of non-coding variants generated by WGS for clinical use do not yet exist.[53] Consequently, few clinically non-coding variants are currently known, and we did not identify any of them in this analysis. However, evidence of regulatory elements that influence the expression of any given gene is accumulating,[54] and high-throughput functional assays for studying them provide the opportunity to define diagnostically significant variants affecting CPGs.[55] If this process were able to elucidate clinically relevant variants, the case for WGS as a first-line investigative tool would become more compelling.

In summary, we have demonstrated that the application of comprehensive CPG testing to a cohort of previously investigated MPT-affected individuals resulted in the detection of multiple pathogenic variants with relevance to the management of those individuals and their relatives. The finding that comprehensive genetic analysis of MPT-affected individuals can frequently result in the identification of pathogenic CPG variants that cannot automatically be attributed as causative for the observed MPT clinical phenotype has important implications both for clinical practice and for future research into the phenotypic consequences of germline CPG variants. Summing together variant detection rates from a previous series of MPT-affected individuals ascertained in a similar manner and the present results suggests that first-line application of WGS (or other strategies for comprehensive CPG variant detection) to a clinical-genetics-referral-based cohort of MPT-affected individuals would detect a deleterious mutation in about a third of individuals, a large proportion of whom would not have a family history of cancer in a first-degree relative.

Accession Numbers

The whole-genome sequencing data reported in this paper have been deposited in the European Genome-phenome Archive under EGA: EGAD00001004088 as a subset of the NIHR BioResource Rare Disease Consortium data.

Supplemental Data

Supplemental Data include two figures and seven tables and can be found with this article online at https://doi.org/10.1016/j.ajhg.2018.04.013.

Consortia

Members of the NIHR BioResource Rare Diseases Consortium include Timothy Aitman, Hana Alachkar, Sonia Ali, Louise Allen, David Allsup, Gautum Ambegaonkar, Julie Anderson, Richard Antrobus, Ruth Armstrong, Gavin Arno, Gururaj Arumugakani, Sofie Ashford, William Astle, Antony Attwood, Steve Austin, Chiara Bacchelli, Tamam Bakchoul, Tadbir K. Bariana, Helen Baxendale, David Bennett, Claire Bethune, Shahnaz Bibi, Maria Bitner-Glindzicz, Marta Bleda, Harm Boggard, Paula Bolton-Maggs, Claire Booth, John R. Bradley, Angie Brady, Matthew Brown, Michael Browning, Christine Bryson, Siobhan Burns, Paul Calleja, Natalie Canham, Jenny Carmichael, Keren Carss, Mark Caulfield, Elizabeth Chalmers, Anita Chandra, Patrick Chinnery, Manali Chitre, Colin Church, Emma Clement, Naomi Clements-Brod, Virginia Clowes, Gerry Coghlan, Peter Collins, Victoria Cookson, Nichola Cooper, Paul Corris, Amanda Creaser-Myers, Rosa DaCosta, Louise Daugherty, Sophie Davies, John Davis, Minka De Vries, Patrick Deegan, Sri V.V. Deevi, Charu Deshpande, Lisa Devlin, Eleanor Dewhurst, Peter Dixon, Rainer Doffinger, Natalie Dormand, Elizabeth Drewe, David Edgar, William Egner, Wendy N. Erber, Marie Erwood, Marie Erwood, Tamara Everington, Remi Favier, Helen Firth, Debra Fletcher, Frances Flinter, Amy Frary, Kathleen Freson, Bruce Furie, Abigail Furnell, Daniel Gale, Alice Gardham, Michael Gattens, Neeti Ghali, Pavandeep K. Ghataorhe, Rohit Ghurye, Simon Gibbs, Kimberley Gilmour, Paul Gissen, Sarah Goddard, Keith Gomez, Pavel Gordins, Stefan Graf, Stefan Gräf, Daniel Greene, Alan Greenhalgh, Andreas Greinacher, Sofia Grigoriadou, Detelina Grozeva, Scott Hackett, Charaka Hadinnapola, Rosie Hague, Matthias Haimel, Csaba Halmagyi, Tracey Hammerton, Daniel Hart, Grant Hayman, Johan W.M. Heemskerk, Robert Henderson, Anke Hensiek, Yvonne Henskens, Archana Herwadkar, Simon Holden, Muriel Holder, Susan Holder, Fengyuan Hu, Anna Huis in't Veld, Aarnoud Huissoon, Marc Humbert, Jane Hurst, Roger James, Stephen Jolles, Dragana Josifova, Rashid Kazmi, David Keeling, Peter Kelleher, Anne M. Kelly, Fiona Kennedy, David Kiely, Nathalie Kingston, Ania Koziell, Deepa Krishnakumar, Taco W. Kuijpers, Taco Kuijpers, Dinakantha Kumararatne, Manju Kurian, Michael A. Laffan, Michele P. Lambert, Hana Lango Allen, Hana Lango-Allen, Allan Lawrie, Sara Lear, Melissa Lees,

Claire Lentaigne, Ri Liesner, Rachel Linger, Hilary Longhurst, Lorena Lorenzo, Eleni Louka, Rajiv Machado, Rob Mackenzie Ross, Robert MacLaren, Eamonn Maher, Jesmeen Maimaris, Sarah Mangles, Ania Manson, Rutendo Mapeta, Hugh S. Markus, Jennifer Martin, Larahmie Masati, Mary Mathias, Vera Matser, Anna Maw, Elizabeth McDermott, Coleen McJannet, Stuart Meacham, Sharon Meehan, Karyn Megy, Sarju Mehta, Michel Michaelides, Carolyn M. Millar, Shahin Moledina, Anthony Moore, Nicholas Morrell, Andrew Mumford, Sai Murng, Elaine Murphy, Sergey Nejentsev, Sadia Noorani, Paquita Nurden, Eric Oksenhendler, Shokri Othman, Willem H. Ouwehand, Willem H. Ouwehand, Sofia Papadia, Soo-Mi Park, Alasdair Parker, John Pasi, Chris Patch, Joan Paterson, Jeanette Payne, Andrew Peacock, Kathelijne Peerlinck, Christopher J. Penkett, Joanna Pepke-Zaba, David Perry, David J. Perry, Val Pollock, Gary Polwarth, Mark Ponsford, Waseem Qasim, Isabella Quinti, Stuart Rankin, Julia Rankin, F. Lucy Raymond, Paula Rayner-Matthews, Karola Rehnstrom, Evan Reid, Christopher J. Rhodes, Michael Richards, Sylvia Richardson, Alex Richter, Irene Roberts, Matthew Rondina, Elisabeth Rosser, Catherine Roughley, Noémi Roy, Kevin Rue-Albrecht, Crina Samarghitean, Alba Sanchis-Juan, Richard Sandford, Saikat Santra, Ravishankar Sargur, Sinisa Savic, Gwen Schotte, Sol Schulman, Harald Schulze, Richard Scott, Marie Scully, Suranjith Seneviratne, Carrock Sewell, Olga Shamardina, Debbie Shipley, Ilenia Simeoni, Suthesh Sivapalaratnam, Kenneth G.C. Smith, Aman Sohal, Laura Southgate, Simon Staines, Emily Staples, Hannah Stark, Hans Stauss, Penelope Stein, Jonathan Stephens, Kathleen Stirrups, Sophie Stock, Jay Suntharalingam, Kate Talks, Yvonne Tan, Jecko Thachil, James Thaventhiran, Ellen Thomas, Moira Thomas, Dorothy Thompson, Adrian Thrasher, Marc Tischkowitz, Catherine Titterton, Cheng-Hock Toh, Mark Toshner, Carmen Treacy, Richard Trembath, Salih Tuna, Wojciech Turek, Ernest Turro, Chris Van Geet, Marijke Veltman, Julie Vogt, Julie von Ziegenweldt, Anton Vonk Noordegraaf, Emma Wakeling, Ivy Wanjiku, Timothy Q. Warner, Evangeline Wassmer, Hugh Watkins, Christopher Watt, Andrew Webster, Steve Welch, Sarah Westbury, John Wharton, Deborah Whitehorn, Martin Wilkins, Lisa Willcocks, Catherine Williamson, Geoffrey Woods, Geoff Woods, John Wort, Nigel Yeatman, Patrick Yong, Tim Young, and Ping Yu.

Acknowledgments

J.W. is supported by a Clinical Research Training Fellowship from the Cancer Research UK Cambridge Cancer Centre. Funding for the National Institute for Health Research (NIHR) BioResource Rare Diseases project was provided by the NIHR (grant number RG65966). E.R.M. acknowledges support from the European Research Council (Advanced Researcher Award), the NIHR (Senior Investigator Award and Cambridge NIHR Biomedical Research Centre), the Cancer Research UK Cambridge Cancer Centre, and the Medical Research Council (Infrastructure Award). The University of Cambridge has received salary support for E.R.M. from the National Health Service (NHS) in the East of England through the Clinical Academic Reserve. The views expressed herein are those of the authors and not necessarily those of the NHS or the Department of Health. D.G.E. is an NIHR Senior Investigator and is supported by the NIHR Manchester Biomedical Research Centre.

Declaration of Interests

D.G.E. received travel grants from Astrazeneca and AmGen.

Received: December 6, 2017
Accepted: April 25, 2018
Published: June 14, 2018

Web Resources

ClinVar, https://www.ncbi.nlm.nih.gov/clinvar/
Ensembl, http://www.ensembl.org
European Genome-phenome Archive, https://ega-archive.org/
ExAC Browser, http://exac.broadinstitute.org/
GenBank, https://www.ncbi.nlm.nih.gov/genbank/
OMIM, https://www.omim.org/
UCSC Genome Browser, https://genome.ucsc.edu/

References

1. Rahman, N. (2014). Realizing the promise of cancer predisposition genes. Nature *505*, 302–308.
2. Gonzalez, K.D., Noltner, K.A., Buzin, C.H., Gu, D., Wen-Fong, C.Y., Nguyen, V.Q., Han, J.H., Lowstuter, K., Longmate, J., Sommer, S.S., and Weitzel, J.N. (2009). Beyond Li Fraumeni Syndrome: clinical characteristics of families with p53 germline mutations. J. Clin. Oncol. *27*, 1250–1256.
3. Metcalfe, K.A., Lynch, H.T., Ghadirian, P., Tung, N., Olivotto, I.A., Foulkes, W.D., Warner, E., Olopade, O., Eisen, A., Weber, B., et al. (2005). The risk of ovarian cancer after breast cancer in BRCA1 and BRCA2 carriers. Gynecol. Oncol. *96*, 222–226.
4. Travis, L.B. (2006). The epidemiology of second primary cancers. Cancer Epidemiol. Biomarkers Prev. *15*, 2020–2026.
5. Carss, K.J., Arno, G., Erwood, M., Stephens, J., Sanchis-Juan, A., Hull, S., Megy, K., Grozeva, D., Dewhurst, E., Malka, S., et al.; NIHR-BioResource Rare Diseases Consortium (2017). Comprehensive rare variant analysis via whole-genome sequencing to determine the molecular pathology of inherited retinal disease. Am. J. Hum. Genet. *100*, 75–90.
6. Belkadi, A., Bolze, A., Itan, Y., Cobat, A., Vincent, Q.B., Antipenko, A., Shang, L., Boisson, B., Casanova, J.-L., and Abel, L. (2015). Whole-genome sequencing is more powerful than whole-exome sequencing for detecting exome variants. Proc. Natl. Acad. Sci. USA *112*, 5473–5478.
7. Suzuki, T., Tsurusaki, Y., Nakashima, M., Miyake, N., Saitsu, H., Takeda, S., and Matsumoto, N. (2014). Precise detection of chromosomal translocation or inversion breakpoints by whole-genome sequencing. J. Hum. Genet. *59*, 649–654.
8. International Agency for Research on Cancer (2004). International rules for multiple primary cancers, third edition (ICD-O).
9. Roller, E., Ivakhno, S., Lee, S., Royce, T., and Tanner, S. (2016). Canvas: versatile and scalable detection of copy number variants. Bioinformatics *32*, 2375–2377.
10. Chen, X., Schulz-Trieglaff, O., Shaw, R., Barnes, B., Schlesinger, F., Källberg, M., Cox, A.J., Kruglyak, S., and Saunders, C.T. (2016). Manta: rapid detection of structural variants and indels for germline and cancer sequencing applications. Bioinformatics *32*, 1220–1222.
11. Li, H., and Durbin, R. (2009). Fast and accurate short read alignment with Burrows-Wheeler transform. Bioinformatics *25*, 1754–1760.
12. Li, H., Handsaker, B., Wysoker, A., Fennell, T., Ruan, J., Homer, N., Marth, G., Abecasis, G., Durbin, R.; and 1000 Genome

Project Data Processing Subgroup (2009). The Sequence Alignment/Map format and SAMtools. Bioinformatics *25*, 2078–2079.

13. McKenna, A., Hanna, M., Banks, E., Sivachenko, A., Cibulskis, K., Kernytsky, A., Garimella, K., Altshuler, D., Gabriel, S., Daly, M., and DePristo, M.A. (2010). The Genome Analysis Toolkit: a MapReduce framework for analyzing next-generation DNA sequencing data. Genome Res. *20*, 1297–1303.
14. DePristo, M.A., Banks, E., Poplin, R., Garimella, K.V., Maguire, J.R., Hartl, C., Philippakis, A.A., del Angel, G., Rivas, M.A., Hanna, M., et al. (2011). A framework for variation discovery and genotyping using next-generation DNA sequencing data. Nat. Genet. *43*, 491–498.
15. McLaren, W., Gil, L., Hunt, S.E., Riat, H.S., Ritchie, G.R.S., Thormann, A., Flicek, P., and Cunningham, F. (2016). The Ensembl Variant Effect Predictor. Genome Biol. *17*, 122.
16. Weren, R.D.A., Ligtenberg, M.J.L., Kets, C.M., de Voer, R.M., Verwiel, E.T.P., Spruijt, L., van Zelst-Stams, W.A.G., Jongmans, M.C., Gilissen, C., Hehir-Kwa, J.Y., et al. (2015). A germline homozygous mutation in the base-excision repair gene NTHL1 causes adenomatous polyposis and colorectal cancer. Nat. Genet. *47*, 668–671.
17. Jafri, M., Wake, N.C., Ascher, D.B., Pires, D.E.V., Gentle, D., Morris, M.R., Rattenberry, E., Simpson, M.A., Trembath, R.C., Weber, A., et al. (2015). Germline mutations in the CDKN2B tumor suppressor gene predispose to renal cell carcinoma. Cancer Discov. *5*, 723–729.
18. Lek, M., Karczewski, K.J., Minikel, E.V., Samocha, K.E., Banks, E., Fennell, T., O'Donnell-Luria, A.H., Ware, J.S., Hill, A.J., Cummings, B.B., et al.; Exome Aggregation Consortium (2016). Analysis of protein-coding genetic variation in 60,706 humans. Nature *536*, 285–291.
19. Auton, A., Brooks, L.D., Durbin, R.M., Garrison, E.P., Kang, H.M., Korbel, J.O., Marchini, J.L., McCarthy, S., McVean, G.A., Abecasis, G.R.; and 1000 Genomes Project Consortium (2015). A global reference for human genetic variation. Nature *526*, 68–74.
20. Landrum, M.J., Lee, J.M., Benson, M., Brown, G., Chao, C., Chitipiralla, S., Gu, B., Hart, J., Hoffman, D., Hoover, J., et al. (2016). ClinVar: public archive of interpretations of clinically relevant variants. Nucleic Acids Res. *44* (D1), D862–D868.
21. Stenson, P.D., Ball, E.V., Mort, M., Phillips, A.D., Shiel, J.A., Thomas, N.S.T., Abeysinghe, S., Krawczak, M., and Cooper, D.N. (2003). Human Gene Mutation Database (HGMD): 2003 update. Hum. Mutat. *21*, 577–581.
22. Kircher, M., Witten, D.M., Jain, P., O'Roak, B.J., Cooper, G.M., and Shendure, J. (2014). A general framework for estimating the relative pathogenicity of human genetic variants. Nat. Genet. *46*, 310–315.
23. University Medical Center Groningen. FaCD Online: Familial cancer database. http://www.facd.info.
24. Robinson, J.T., Thorvaldsdóttir, H., Winckler, W., Guttman, M., Lander, E.S., Getz, G., and Mesirov, J.P. (2011). Integrative genomics viewer. Nat. Biotechnol. *29*, 24–26.
25. Richards, S., Aziz, N., Bale, S., Bick, D., Das, S., Gastier-Foster, J., Grody, W.W., Hegde, M., Lyon, E., Spector, E., et al.; ACMG Laboratory Quality Assurance Committee (2015). Standards and guidelines for the interpretation of sequence variants: a joint consensus recommendation of the American College of Medical Genetics and Genomics and the Association for Molecular Pathology. Genet. Med. *17*, 405–424.
26. Zarrei, M., MacDonald, J.R., Merico, D., and Scherer, S.W. (2015). A copy number variation map of the human genome. Nat. Rev. Genet. *16*, 172–183.
27. Smedley, D., Haider, S., Durinck, S., Pandini, L., Provero, P., Allen, J., Arnaiz, O., Awedh, M.H., Baldock, R., Barbiera, G., et al. (2015). The BioMart community portal: an innovative alternative to large, centralized data repositories. Nucleic Acids Res. *43* (W1), W589–W598.
28. R Core Team (2017). R: A language and environment for statistical computing (R Foundation for Statistical Computing).
29. Casey, R.T., Warren, A.Y., Martin, J.E., Challis, B.G., Rattenberry, E., Whitworth, J., Andrews, K.A., Roberts, T., Clark, G.R., West, H., et al. (2017). Clinical and Molecular Features of Renal and Pheochromocytoma/Paraganglioma Tumor Association Syndrome (RAPTAS): Case Series and Literature Review. J. Clin. Endocrinol. Metab. *102*, 4013–4022.
30. Comino-Méndez, I., Gracia-Aznárez, F.J., Schiavi, F., Landa, I., Leandro-García, L.J., Letón, R., Honrado, E., Ramos-Medina, R., Caronia, D., Pita, G., et al. (2011). Exome sequencing identifies MAX mutations as a cause of hereditary pheochromocytoma. Nat. Genet. *43*, 663–667.
31. Whitworth, J., Hoffman, J., Chapman, C., Ong, K.R., Lalloo, F., Evans, D.G., and Maher, E.R. (2015). A clinical and genetic analysis of multiple primary cancer referrals to genetics services. Eur. J. Hum. Genet. *23*, 581–587.
32. Thompson, D., Duedal, S., Kirner, J., McGuffog, L., Last, J., Reiman, A., Byrd, P., Taylor, M., and Easton, D.F. (2005). Cancer risks and mortality in heterozygous ATM mutation carriers. J. Natl. Cancer Inst. *97*, 813–822.
33. Weischer, M., Bojesen, S.E., Tybjaerg-Hansen, A., Axelsson, C.K., and Nordestgaard, B.G. (2007). Increased risk of breast cancer associated with CHEK2*1100delC. J. Clin. Oncol. *25*, 57–63.
34. Rahman, N., Seal, S., Thompson, D., Kelly, P., Renwick, A., Elliott, A., Reid, S., Spanova, K., Barfoot, R., Chagtai, T., et al.; Breast Cancer Susceptibility Collaboration (UK) (2007). PALB2, which encodes a BRCA2-interacting protein, is a breast cancer susceptibility gene. Nat. Genet. *39*, 165–167.
35. Antoniou, A.C., Casadei, S., Heikkinen, T., Barrowdale, D., Pylkäs, K., Roberts, J., Lee, A., Subramanian, D., De Leeneer, K., Fostira, F., et al. (2014). Breast-cancer risk in families with mutations in PALB2. N. Engl. J. Med. *371*, 497–506.
36. Lalloo, F., Varley, J., Ellis, D., Moran, A., O'Dair, L., Pharoah, P., Evans, D.G.R.; and Early Onset Breast Cancer Study Group (2003). Prediction of pathogenic mutations in patients with early-onset breast cancer by family history. Lancet *361*, 1101–1102.
37. Schrader, K.A., Cheng, D.T., Joseph, V., Prasad, M., Walsh, M., Zehir, A., Ni, A., Thomas, T., Benayed, R., Ashraf, A., et al. (2016). Germline variants in targeted tumor sequencing using matched normal DNA. JAMA Oncol. *2*, 104–111.
38. Mandelker, D., Zhang, L., Kemel, Y., Stadler, Z.K., Joseph, V., Zehir, A., Pradhan, N., Arnold, A., Walsh, M.F., Li, Y., et al. (2017). Mutation detection in patients with advanced cancer by universal sequencing of cancer-related genes in tumor and normal DNA vs guideline-based germline testing. JAMA *318*, 825–835.
39. Zhang, J., Walsh, M.F., Wu, G., Edmonson, M.N., Gruber, T.A., Easton, J., Hedges, D., Ma, X., Zhou, X., Yergeau, D.A., et al. (2015). Germline Mutations in Predisposition Genes in Pediatric Cancer. N. Engl. J. Med. *373*, 2336–2346.

40. Parsons, D.W., Roy, A., Yang, Y., Wang, T., Scollon, S., Bergstrom, K., Kerstein, R.A., Gutierrez, S., Petersen, A.K., Bavle, A., et al. (2016). Diagnostic yield of clinical tumor and germline whole-exome sequencing for children with solid tumors. JAMA Oncol. *2*, 616–624.
41. Mody, R.J., Wu, Y.M., Lonigro, R.J., Cao, X., Roychowdhury, S., Vats, P., Frank, K.M., Prensner, J.R., Asangani, I., Palanisamy, N., et al. (2015). Integrative clinical sequencing in the management of refractory or relapsed cancer in youth. JAMA *314*, 913–925.
42. Pearlman, R., Frankel, W.L., Swanson, B., Zhao, W., Yilmaz, A., Miller, K., Bacher, J., Bigley, C., Nelsen, L., Goodfellow, P.J., et al.; Ohio Colorectal Cancer Prevention Initiative Study Group (2017). Prevalence and spectrum of germline cancer susceptibility gene mutations among patients with early-onset colorectal cancer. JAMA Oncol. *3*, 464–471.
43. Cybulski, C., Górski, B., Huzarski, T., Masojć, B., Mierzejewski, M., Dębniak, T., Teodorczyk, U., Byrski, T., Gronwald, J., Matyjasik, J., et al. (2004). CHEK2 is a multiorgan cancer susceptibility gene. Am. J. Hum. Genet. *75*, 1131–1135.
44. Aoude, L.G., Xu, M., Zhao, Z.Z., Kovacs, M., Palmer, J.M., Johansson, P., Symmons, J., Trent, J.M., Martin, N.G., Montgomery, G.W., et al. (2014). Assessment of PALB2 as a candidate melanoma susceptibility gene. PLoS ONE *9*, e100683.
45. Swift, M., Morrell, D., Massey, R.B., and Chase, C.L. (1991). Incidence of cancer in 161 families affected by ataxia-telangiectasia. N. Engl. J. Med. *325*, 1831–1836.
46. Tomlinson, I.P.M., Alam, N.A., Rowan, A.J., Barclay, E., Jaeger, E.E.M., Kelsell, D., Leigh, I., Gorman, P., Lamlum, H., Rahman, S., et al.; Multiple Leiomyoma Consortium (2002). Germline mutations in FH predispose to dominantly inherited uterine fibroids, skin leiomyomata and papillary renal cell cancer. Nat. Genet. *30*, 406–410.
47. Castro-Vega, L.J., Buffet, A., De Cubas, A.A., Cascón, A., Menara, M., Khalifa, E., Amar, L., Azriel, S., Bourdeau, I., Chabre, O., et al. (2014). Germline mutations in FH confer predisposition to malignant pheochromocytomas and paragangliomas. Hum. Mol. Genet. *23*, 2440–2446.
48. Clark, G.R., Sciacovelli, M., Gaude, E., Walsh, D.M., Kirby, G., Simpson, M.A., Trembath, R.C., Berg, J.N., Woodward, E.R., Kinning, E., et al. (2014). Germline FH mutations presenting with pheochromocytoma. J. Clin. Endocrinol. Metab. *99*, E2046–E2050.
49. Coughlin, E.M., Christensen, E., Kunz, P.L., Krishnamoorthy, K.S., Walker, V., Dennis, N.R., Chalmers, R.A., Elpeleg, O.N., Whelan, D., Pollitt, R.J., et al. (1998). Molecular analysis and prenatal diagnosis of human fumarase deficiency. Mol. Genet. Metab. *63*, 254–262.
50. Whitworth, J., Skytte, A.-B., Sunde, L., Lim, D.H., Arends, M.J., Happerfield, L., Frayling, I.M., van Minkelen, R., Woodward, E.R., Tischkowitz, M.D., and Maher, E.R. (2016). Multilocus inherited neoplasia alleles syndrome: A case series and review. JAMA Oncol. *2*, 373–379.
51. National Human Genome Research Institute (2016). The Cost of Sequencing a Human Genome, https://www.genome.gov/sequencingcosts/.
52. Tattini, L., D'Aurizio, R., and Magi, A. (2015). Detection of genomic structural variants from next-generation sequencing data. Front. Bioeng. Biotechnol. *3*, 92.
53. Feero, W.G. (2014). Clinical application of whole-genome sequencing: proceed with care. JAMA *311*, 1017–1019.
54. Dunham, I., Kundaje, A., Aldred, S.F., Collins, P.J., Davis, C.A., Doyle, F., Epstein, C.B., Frietze, S., Harrow, J., Kaul, R., et al.; ENCODE Project Consortium (2012). An integrated encyclopedia of DNA elements in the human genome. Nature *489*, 57–74.
55. Ipe, J., Swart, M., Burgess, K.S., and Skaar, T.C. (2017). High-throughput assays to assess the functional impact of genetic variants: A road towards genomic-driven medicine. Clin. Transl. Sci. *10*, 67–77.

The American Journal of Human Genetics, Volume *103*

Supplemental Data

Comprehensive Cancer-Predisposition Gene Testing in an Adult Multiple Primary Tumor Series Shows a Broad Range of Deleterious Variants and Atypical Tumor Phenotypes

James Whitworth, Philip S. Smith, Jose-Ezequiel Martin, Hannah West, Andrea Luchetti, Faye Rodger, Graeme Clark, Keren Carss, Jonathan Stephens, Kathleen Stirrups, Chris Penkett, Rutendo Mapeta, Sofie Ashford, Karyn Megy, Hassan Shakeel, Munaza Ahmed, Julian Adlard, Julian Barwell, Carole Brewer, Ruth T. Casey, Ruth Armstrong, Trevor Cole, Dafydd Gareth Evans, Florentia Fostira, Lynn Greenhalgh, Helen Hanson, Alex Henderson, Jonathan Hoffman, Louise Izatt, Ajith Kumar, Ava Kwong, Fiona Lalloo, Kai Ren Ong, Joan Paterson, Soo-Mi Park, Rakefet Chen-Shtoyerman, Claire Searle, Lucy Side, Anne-Bine Skytte, Katie Snape, Emma R. Woodward, NIHR BioResource Rare Diseases Consortium, Marc D. Tischkowitz, and Eamonn R. Maher

Supplemental Data

Supplemental Figures

Figure S1 - SNV/indel workflow

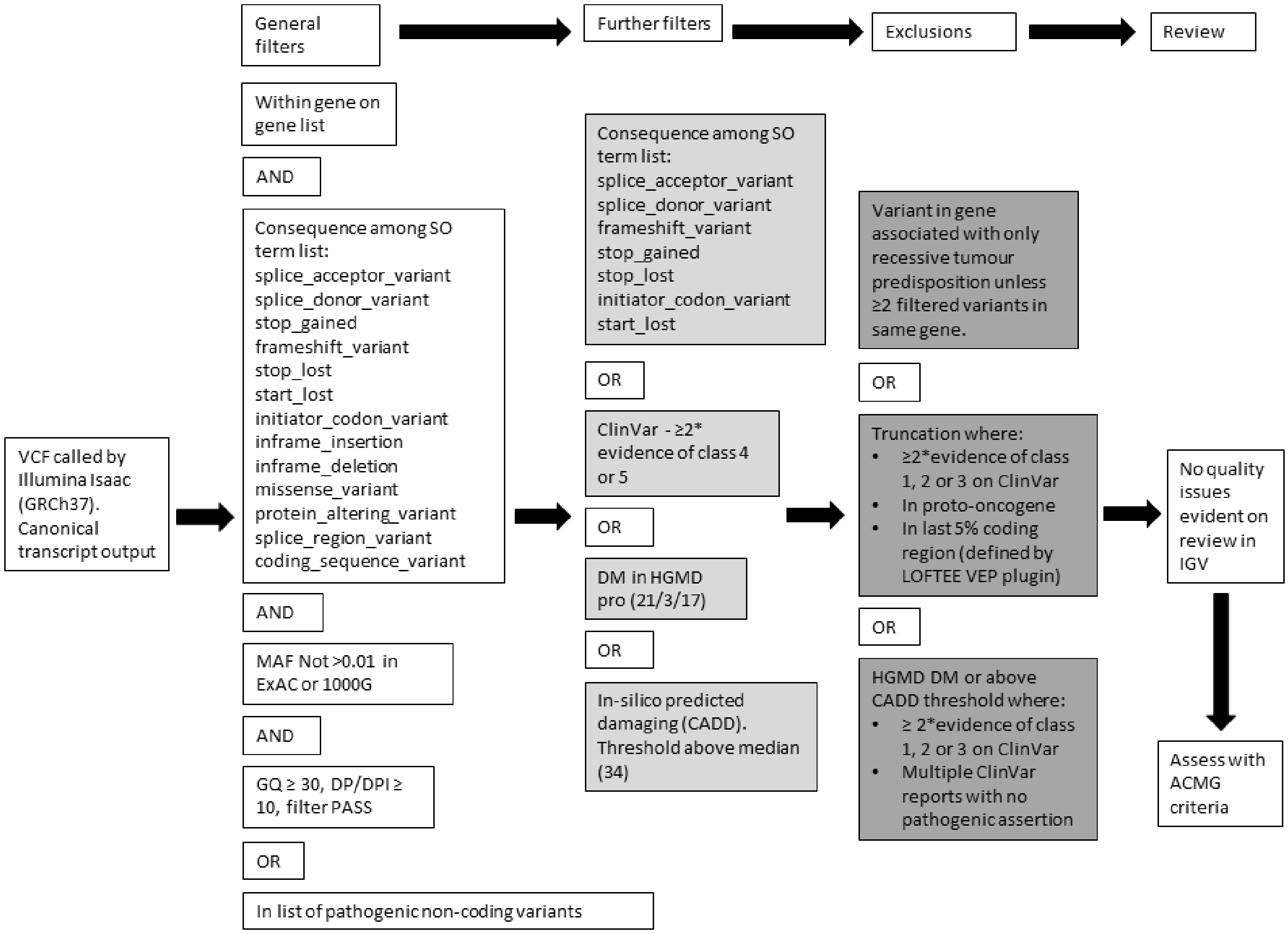

Figure S2 - Structural variant workflow

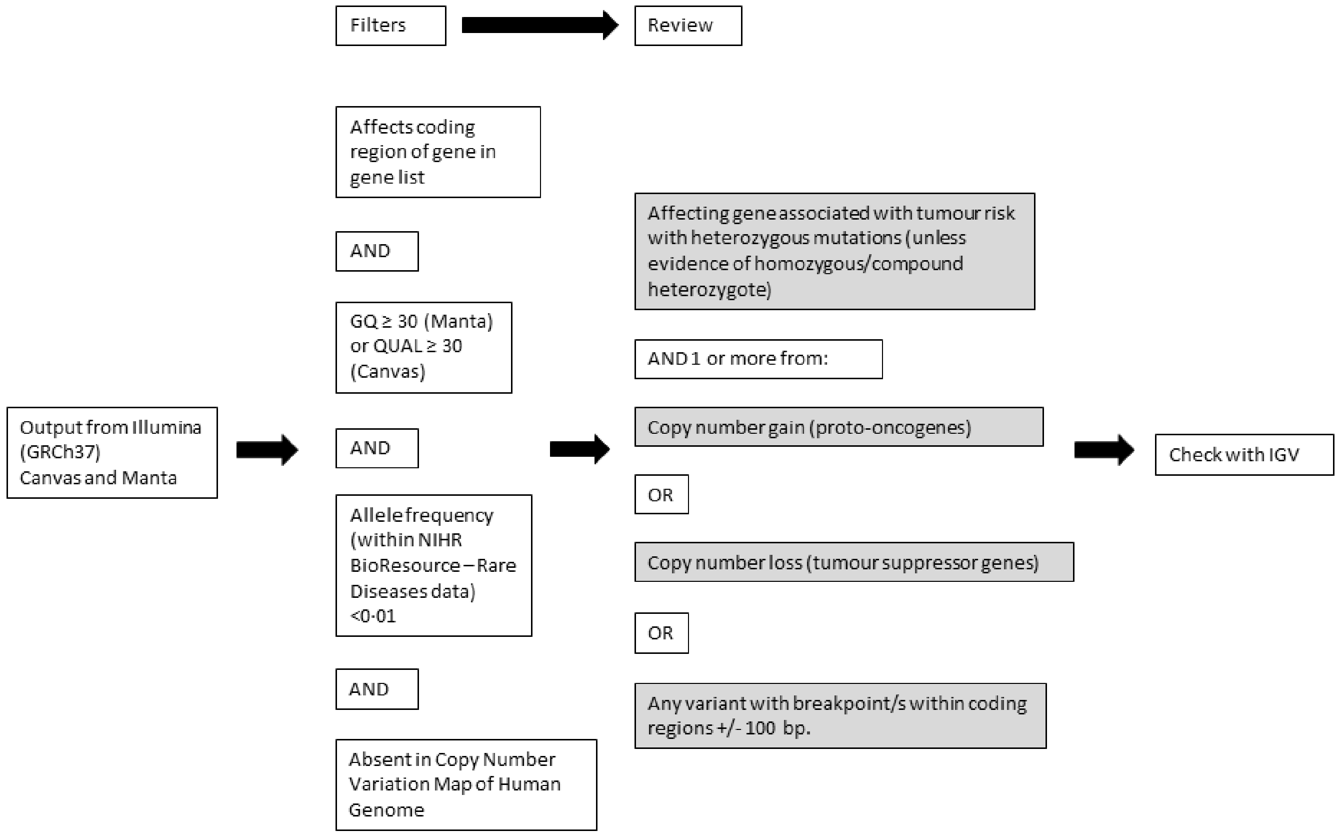

Supplemental tables

Table S2 – Genes sequenced by Illumina TruSight Cancer panel on list of 83 analyzed genes

AIP	*DICER1*	*MLH1*	*RUNX1*
ALK	*EGFR*	*MSH2*	*SDHAF2*
APC	*EPCAM*	*MSH6*	*SDHB*
ATM	*ERCC2*	*MUTYH*	*SDHC*
BAP1	*ERCC3*	*NF1*	*SDHD*
BMPR1A	*ERCC4*	*NF2*	*SMAD4*
BRCA1	*ERCC5*	*PALB2*	*SMARCB1*
BRCA2	*EXT1*	*PHOX2B*	*STK11*
BRIP1	*EXT2*	*PMS2*	*SUFU*
CDC73	*FH*	*PRKAR1A*	*TMEM127*
CDH1	*FLCN*	*PTCH1*	*TP53*
CDK4	*GATA2*	*PTEN*	*TSC1*
CDKN2A	*HNF1A*	*RAD51C*	*TSC2*
CEBPA	*KIT*	*RAD51D*	*VHL*
CHEK2	*MAX*	*RB1*	*WT1*
CYLD	*MEN1*	*RET*	*XPA*
DDB2	*MET*	*RHBDF2*	*XPC*

Table S7 – Comparison of tumor combinations in series vs East Anglia Registry 2009-2014.

Cancer A	Cancer B	Data source	Combination count	% total	Combination type makes up ≥1% total in MPT and EA	Difference in proportion of total MPT vs EA (%)	χ^2 p-value (MPT vs EA)
Breast	Colorectal	MPT	29	5.5	YES	2.7	0.02145
Breast	Ovary	MPT	23	4.4	YES	2.5	0.01786
Breast	Endometrium	MPT	20	3.8	YES	0.1	0.8852
Breast	NMSC	MPT	19	3.6	YES	-10.2	<0.00001
Breast	Thyroid	MPT	19	3.6	NO	3.2	0.00007
Breast	Hem Lymphoid	MPT	18	3.4	YES (note different classification MPT vs EA)	2	0.02594
Endometrium	Ovary	MPT	17	3.3	YES	1.6	0.1073
Breast	Melanoma	MPT	14	2.7	YES	0.6	0.5397
Breast	CNS Meningioma	MPT	7	1.3	NO (Classified as "Brain" in EA)	0.9	0.06944
Breast	Kidney	MPT	6	1.1	NO	0.9	0.04323
Breast	Lung	MPT	6	1.1	NO	0	0.252
Melanoma	Thyroid	MPT	6	1.1	NO	0.7	0.1204
Breast	NMSC	EA	79	13.8	NO	N/A	N/A
Melanoma	NMSC	EA	64	11.1	NO	N/A	N/A
Hematological	NMSC	EA	29	5.1	NO	N/A	N/A
NMSC	Prostate	EA	26	4.5	NO	N/A	N/A
Breast	Uterus	EA	21	3.7	NO	N/A	N/A
Breast	Melanoma	EA	19	3.3	NO	N/A	N/A
Breast	Ovary	EA	11	1.9	NO	N/A	N/A
Prostate	Renal tract	EA	10	1.7	NO	N/A	N/A
Ovary	Uterus	EA	10	1.7	NO	N/A	N/A
NMSC	Thyroid	EA	9	1.6	NO	N/A	N/A
Breast	Hematological	EA	8	1.4	NO	N/A	N/A
Breast	Colorectal	EA	16	2.8	NO	N/A	N/A
Colorectal	NMSC	EA	14	2.4	NO	N/A	N/A
Breast	Cervix	EA	7	1.2	NO	N/A	N/A
Hematological	Prostate	EA	6	1.0	NO	N/A	N/A
NMSC	Ovary	EA	6	1.0	NO	N/A	N/A
NMSC	Uterus	EA	6	1.0	NO	N/A	N/A

Only tumors occurring before age 60 included for consistency with registry data as obtained.

MPT – Multiple primary tumor series (data analyzed in this study). EA – East Anglia Registry Data.

CNS – Central nervous system, Hem – Hematological, NMSC - Non-melanoma skin cancer (includes basal cell carcinoma and squamous cell carcinoma).

Supplemental References

1. Rahman, N. (2014). Realizing the promise of cancer predisposition genes. Nature 505, 302–308.

2. Finn, R.D., Coggill, P., Eberhardt, R.Y., Eddy, S.R., Mistry, J., Mitchell, A.L., Potter, S.C., Punta, M., Qureshi, M., Sangrador-Vegas, A., et al. (2015). The Pfam protein families database: towards a more sustainable future. Nucleic Acids Res. 44, D279–D285.

3. James Kent, W., Sugnet, C.W., Furey, T.S., Roskin, K.M., Pringle, T.H., Zahler, A.M., and Haussler, D. (2002). The human genome browser at UCSC. Genome Res. 12, 996–1006.
